# Systematic Dissection of the *Agrobacterium* Type VI Secretion System Reveals Machinery and Secreted Components for Subcomplex Formation

**DOI:** 10.1371/journal.pone.0067647

**Published:** 2013-07-05

**Authors:** Jer-Sheng Lin, Lay-Sun Ma, Erh-Min Lai

**Affiliations:** 1 Institute of Plant and Microbial Biology, Academia Sinica, Taipei, Taiwan; 2 Molecular and Biological Agricultural Sciences Program, Taiwan International Graduate Program, National Chung-Hsing University and Academia Sinica, Taipei, Taiwan; 3 Graduate Institute of Biotechnology, National Chung-Hsing University, Taichung, Taiwan; Monash University, Australia

## Abstract

The type VI secretion system (T6SS) is widely distributed in pathogenic *Proteobacteria*. Sequence and structural analysis of T6SS reveals a resemblance to the T4 bacteriophage tail, in which an outer sheath structure contracts an internal tube for injecting nucleic acid into bacterial cells. However, the molecular details of how this phage tail-like T6SS structure is assembled *in vivo* and executed for exoprotein or effector secretion remain largely unknown. Here, we used a systematic approach to identify T6SS machinery and secreted components and investigate the interaction among the putative sheath and tube components of *Agrobacterium tumefaciens*. We showed that 14 T6SS components play essential roles in the secretion of the T6SS hallmark exoprotein Hcp. In addition, we discovered a novel T6SS exoprotein, Atu4347, that is dispensable for Hcp secretion. Interestingly, Atu4347 and the putative tube components, Hcp and VgrG, are mainly localized in the cytoplasm but also detected on the bacterial surface. Atu4342 (TssB) and Atu4341 (TssC_41_) interact with and stabilize each other, which suggests that they are functional orthologs of the sheath components TssB (VipA) and TssC (VipB), respectively. Importantly, TssB interacts directly with the three exoproteins (Hcp, VgrG, and Atu4347), in which Hcp also interacts directly with VgrG-1 on co-purification from *Escherichia coli*. Further co-immunoprecipitation and pulldown assays revealed these subcomplex(es) in *A. tumefaciens* and thereby support T6SS functioning as a contractile phage tail-like structure.

## Introduction

Protein secretion systems play central roles in export or import of macromolecules across the cell envelope in bacteria. Gram-negative bacteria have 6 types of specialized protein secretion systems (type I to VI secretion system [T1SS to T6SS]) [1]; T6SS is the most recently described [2] and widespread in bacteria [3]–[6]. T6SS is tightly regulated and has a variety of biological functions such as promoting or limiting virulence and cytotoxicity in eukaryotic or bacterial hosts [7]–[10].

A hallmark of a functional T6SS is the secretion of Hcp and/or VgrG to the extracellular milieu. A unique feature of Hcp and VgrG is that they are both secreted exoproteins but are also part of a secretion apparatus [2], [11]–[13]. Growing evidence of structural and sequence analogy of several T6SS components to the T4 bacteriophage tail (baseplate, tail tube and sheath) suggests that the T6SS machinery and phage tail are evolutionarily conserved. Hcp forms a hexameric ring structure with a 4.0-nm internal pore and can stack into a head-to-tail tube *in vitro* [14]–[17]. In addition, VgrG structurally resembles the phage tail spike (gp5)_3_–(gp27)_3_ complex, and Hcp possesses sequence homology to the phage tube gp19 and is structurally similar to gp5 and the tandem tube domain of gp27 [11], [16], [18]. Thus, the Hcp and VgrG exoproteins may assemble into a phage tail-like structure. Overexpression of the TssB–TssC proteins, *Vibrio cholerae* VipA–VipB and *Pseudomonas aeruginosa* HsiB1–HsiC1, in *E. coli* produced a cogwheel-like tubular structure [19]–[21]. Importantly, both a thinner/extended tube with dense interior and thicker/contracted hollow structure were also seen in the cytoplasm of *V. cholerae* cell by electron cryotomography [22] and immunogold labeling by transmission electron microscopy [23]. Furthermore, Mekalanos and colleagues discovered that TssB–TssC tubule structures are highly dynamic and cycle between an extended and contracted conformation [22], a phenomenon also recently reported by other groups [23], [24]. The phage tail sheath of a contractile phage wraps around the tail tube and propels the tail tube toward the target cell interior upon infection [25]. Therefore, the TssB–TssC tubule may wrap around the Hcp tube to form the extended tubule and contract to push the Hcp tube across bacterial membranes from interior cells. However, how the TssB–TssC tubule accommodates the Hcp tube and coordinates with other T6SS components for T6SS exoprotein or substrate secretion across membranes remain unknown.

In contrast to Hcp, which is likely not an effector, some evolved VgrG family proteins have effector activity. The actin cross-linking domain (ACD) of *V. cholerae* VgrG-1 [2], [11], [26], [27] and vegetative insecticidal protein (VIP-2) domain of *Aeromonas hydrophila* VgrG-1 [28] are both responsible for host cell cytotoxicity. In addition, a number of T6SS-secreted effectors were recently found to exhibit interbacterial killing activity in various bacteria [12], [29]–[32]. This interbacterial response or killing activity was also visualized by time-lapse fluorescent microscopy [24], [33], [34], for a new way to detect T6SS activity at the single cell level.

Systematic mutagenesis analysis of the T6SS locus from *Edwardsiella tarda* and *V. cholera*, along with other studies, revealed 13 conserved components of type VI secretion (Tss, nomenclature proposed by Shalom et al. [35]) that are essential for mediating T6SS exoprotein secretion [9], [12], [36]. Among them, TssM (IcmF), TssL (IcmH or DotU), and TagL (SciZ) are integral inner membrane (IM) proteins and form a transmembrane protein complex that interacts with the TssJ (SciN) outer membrane (OM) lipoprotein [37]–[41]. Like most protein secretion systems, T6SS involves ATPases. ClpV1 is an AAA+ ATPase [14], [19] that converts a TssB–TssC tubule structure into smaller complexes [19], [20] and specifically binds to the contracted TssB–TssC tubule for disassembly and cycling [22], [23]. However, TssM functions to recruit Hcp into the TssM–TssL IM complex and powers Hcp secretion via ATP hydrolysis [42]. TssE, with homology to the baseplate gp25, is essential for Hcp secretion [16], [43] and critical for assembly of the TssB–TssC tubule [22].

We previously identified a functional T6SS by secretome analysis [44] and discovered that the T6SS is activated by acidity [45] in *Agrobacterium tumefaciens,* a pathogenic bacterium causing crown gall disease in plants. In this study, we used a systematic approach to dissect the components of the T6SS machinery and secreted exoproteins in *A. tumefaciens*. We discovered a novel T6SS secreted exoprotein, Atu4347, and identified 14 T6SS components that are required for Hcp secretion. We also identified the functional orthologs of the TssB–TssC complex that interacts with three exoproteins, Hcp, VgrG, and Atu4347. Importantly, we discovered interactions among the three exoproteins and their localization in the cytoplasm and on the bacterial surface. We provide the first demonstration of a Hcp–VgrG direct interaction and physical interactions between components of the sheath structure and internal tube to further document T6SS functioning as a contractile phage tail-like structure.

## Results

### The T6SS Gene Cluster Consists of the *imp* and *hcp* Operons Transcribed from Divergent Promoters

**Figure 1:**
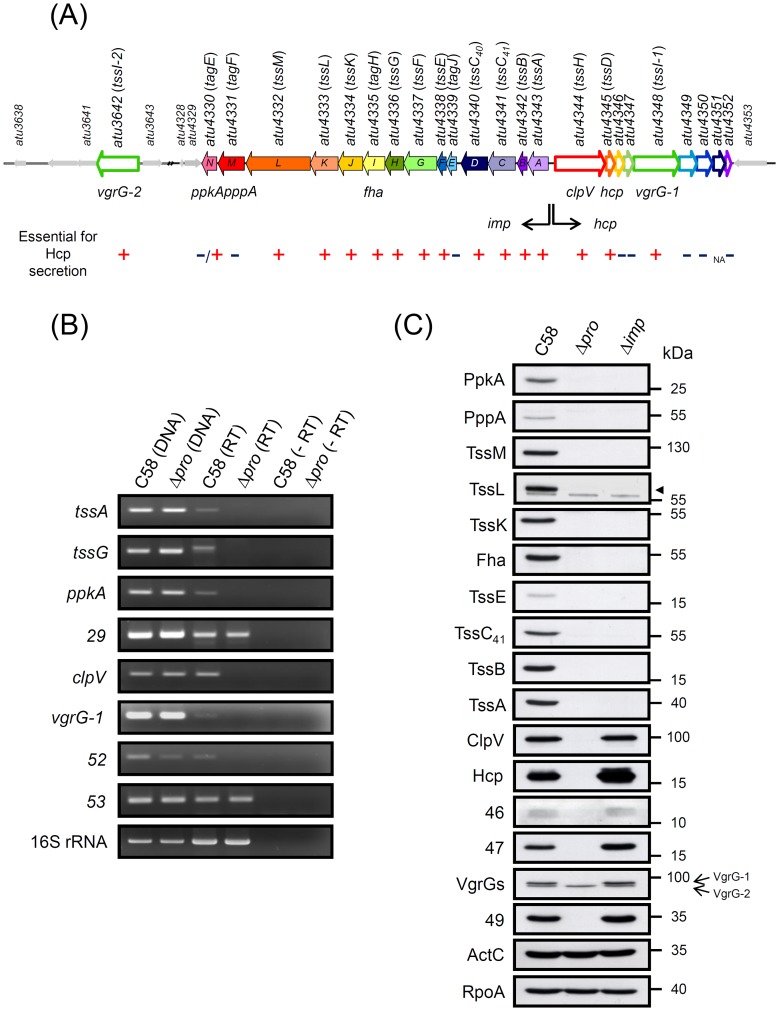
The T6SS gene cluster consists of the *imp* and *hcp* operons transcribed from divergent promoters. (**A**) The *imp* operon consisting of 14 genes (*atu4343* to *atu4330*) is in filled colors and the *hcp* operon encoding nine genes (*atu4344* to *atu4352*) and *atu3642* (*vgrG-2*) encoded elsewhere is in open colors. The genes (indicated with locus names) with homology to conserved T6SS components are designated as *tss* (type VI secretion) or *tag* (type VI secretion-associated gene) based on nomenclature proposed by Shalom et al. [35]. Each of the genes are indicated as essential (+), important but not essential (−/+), dispensable (−) for Hcp secretion, or not analyzed (NA) from results of Hcp secretion assays presented in Figure 2A and Figure S1 in File S1. (**B**) RT-PCR analysis. Total RNA extracted from wild-type *A. tumefaciens* C58 and Δ*pro* strains grown in AB-MES (pH 5.5) for 6 h at 25°C was treated with RNase-free DNase I followed by RT prior to PCR with gene-specific primers. Genomic DNA and total RNA without RT were positive and negative controls, respectively. The 16S rRNA and the genes *atu4329* adjacent to *imp* operon and *atu4353* adjacent to *hcp* operon were internal controls. PCR products were resolved by 2% agarose gel and stained with ethidium bromide with the analyzed genes as indicated. All analyzed genes with *atu* number are shown in abbreviation with their last two numbers (e.g. *atu4329* is shown as *29*). (**C**) Western blot analysis. Total proteins isolated from wild-type C58, Δ*pro*, and Δ*imp* strains grown in the same growth conditions used for RT-PCR were resolved by 10% or 12% Glycine-SDS-PAGE and analyzed by western blot analysis with specific antibodies as indicated. ActC and RpoA were loading controls. The proteins analyzed and the molecular weight markers are indicated on the left and right, respectively, and with arrows when necessary.

T6SS usually exists as one or multiple copies organized in clusters in the genome [3], [5]. In *A. tumefaciens* strain C58, the T6SS gene cluster likely comprises two operons: the *imp* operon, consisting of 14 genes (*atu4343* to *atu4330*), and the *hcp* operon, consisting of nine genes (*atu4344* to *atu4352*), expressed with divergent orientations [44] (Figure 1A). To determine whether the ∼220-bp intergenic region between *atu4343* (*tssA*) and *atu4344* (*clpV*) contains the promoters responsible for the expression of the T6SS genes, we generated a mutant, Δ*pro*, with deletion of this intergenic region. RT-PCR analysis of the selected first, middle, and last genes of the putative *imp* and *hcp* operons revealed the expression of respective transcripts in wild-type C58 but not Δ*pro* (Figure 1B). As internal controls, the transcripts of *atu4329* and *atu4353*, genes adjacent to both operons, and housekeeping 16S rRNA gene were expressed at comparable levels in both C58 and Δ*pro* strains (Figure 1B), which suggests that the deletion of this ∼220-bp intergenic region does not globally affect the expression of genes elsewhere. Similarly, western blot analysis revealed all analyzed proteins encoded from the *imp* and *hcp* operons only in C58 but not Δ*pro*, whereas the levels of VgrG-2, the second VgrG copy encoded outside of this T6SS gene cluster, and two internal controls, citrate transporter ActC [46] and RNA polymerase α-subunit RpoA, were similar in all strains (Figure 1C). Moreover, all examined proteins encoded by the *hcp* operon were expressed in both C58 and Δ*imp*, a mutant with deletion of the entire *imp* operon, so the *imp* operon may not play an essential role in the expression of the *hcp* operon (Figure 1C). Taken together, our data indicate that the *imp* and *hcp* operons are transcribed divergently from promoters located in the intergenic region between *tssA* and *clpV*. This result is consistent with our recent discovery that ChvI response regulator binds to this promoter region in a phosphorylation-dependent manner to activate acid-induced T6SS expression and secretion [45].

### Systematic Mutagenesis Analysis Reveals 14 T6SS Components Required for Hcp Secretion

**Figure 2:**
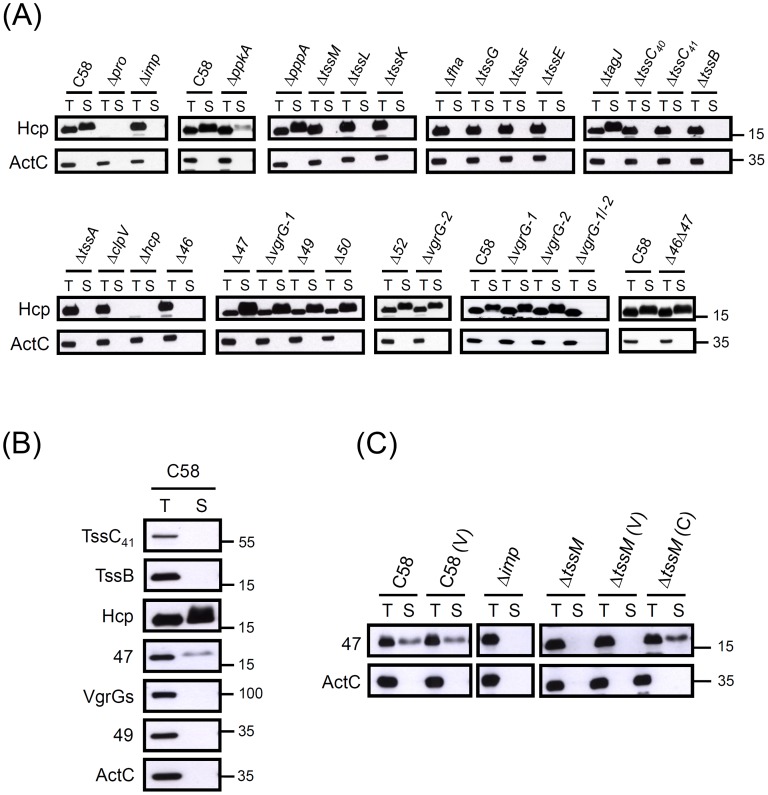
Dissection of the T6SS components required for Hcp secretion and discovery of a novel T6SS-secreted exoprotein, Atu4347. (**A**) Hcp secretion assay. Wild-type *A. tumefaciens* C58, Δ*pro*, Δ*imp*, each of the in-frame deletion mutants, and the Δ*vgrG-1*/−*2* double mutant were analyzed for Hcp secretion. All analyzed genes with *atu* number are shown in abbreviation with their last two numbers (e.g. *atu4346* is shown as *46*). (**B**) Secretion assay of Atu4347 and selected T6SS components. (**C**) Atu4347 is secreted via T6SS in *A. tumefaciens*. Total (T) and secreted (S) proteins isolated from wild-type C58 and various strains grown in AB-MES (pH 5.5) for 6 h at 25°C were separated by 12% Glycine-SDS-PAGE and examined by western blot analysis with specific antibodies as indicated. The secreted proteins were collected from 1 ml (for Hcp) or 2 ml (for Atu4347) of culture medium after removal of bacterial cells by centrifugation and were concentrated by TCA precipitation [44]. The non-secreted protein ActC was an internal control. The proteins analyzed and sizes of molecular weight standards are indicated on the left and right, respectively. The slightly slower migration of secreted Hcp than cellular Hcp is mainly caused by the presence of TCA used for protein precipitated from culture medium [45].

For the 23 genes residing in the T6SS gene cluster, 14 proteins encoded from the *imp* operon are conserved in all or several T6SSs, but only 3 proteins (ClpV, Hcp, VgrG-1) encoded from the *hcp* operon are well conserved (Figure 1A and Table 1). TssM and TssL, two IM components that interact with each other, are essential in mediating Hcp secretion from *A. tumefaciens* [37]. To identify additional T6SS components participating in Hcp secretion, we generated a series of mutants with in-frame deletion in each gene encoded in this gene cluster (except *atu4351*, which could not be deleted) and *vgrG-2* encoded outside of this T6SS gene cluster (Figure 1A). On the basis of their requirement in mediating the secretion of the T6SS hallmark exoprotein Hcp, we then assigned the corresponding gene encoding the T6SS components essential for T6SS secretion. Although Hcp is expressed and secreted into culture medium from wild-type C58 and nine mutants, no Hcp secretion was detected from Δ*imp* and the other 13 mutants (Figure 2A). Except for Δ*hcp*, all Hcp-secretion deficient mutants accumulated intracellular Hcp protein at comparable or slightly higher levels than that of wild-type C58, which suggests their roles as components of T6SS protein channel to mediate Hcp secretion without affecting its expression (Figure 2A). The dispensability of *vgrG-1* for Hcp secretion (Figure 2A) led us to explore whether *vgrG-2* may functionally compensate *vgrG-1* in mediating Hcp secretion in the absence of *vgrG-1*. Indeed, Hcp secretion was normal in the Δ*vgrG-1 *or Δ*vgrG-2* single mutant but blocked in the Δ*vgrG-1/−2* double mutant (Figure 2A). ActC served as a non-secreted periplasmic protein control to ensure the quality of secretion assay.

**Table 1:** Comparison of the type VI secretion components required for Hcp secretion from *Agrobacterium tumefaciens*, *Vibrio cholera* and *Edwardsiella tarda*.

		*A. tumefaciens* a		*V. cholerae* b		*E. tarda* c	
Tss name	Common name	Locus name	Hcp secretion	Locus name	Hcp secretion	Locus name	Hcp secretion
TagE	PpkA	Atu4330/ImpN	−	NA	NA	NA	NA
TagF	PppA	Atu4331/ImpM	−	NA	NA	NA	NA
TssM	IcmF	Atu4332/ImpL	+	VCA0120/VasK	+	EvpO	+
TssL	IcmH	Atu4333/ImpK	+	VCA0115/VasF	+	EvpN	+
TssK		Atu4334/ImpJ	+	VCA0114	+	EvpM	+
TagH	Fha	Atu4335/ImpI	+	VCA0112	+	NA	NA
TssG		Atu4336/ImpH	+	VCA0111	+	EvpG	+
TssF		Atu4337/ImpG	+	VCA0110/VasA	+	EvpF	+
TssE		Atu4338/ImpF	+	VCA0109/gp25 homolog	+	EvpE	+
TagJ		Atu4339/ImpE	−	NA	NA	NA	NA
TssC		Atu4340/ImpD	+	VCA0108/VipB	+	EvpB	+
TssC		Atu4341/ImpC	+	VCA0108/VipB	+	EvpB	+
TssB		Atu4342/ImpB	+	VCA0107/VipA	+	EvpA	+
TssA		Atu4343/ImpA	+	VCA0119	+	EvpK	+
TssH	ClpV	Atu4344	+	VCA0116	+	EvpH	+
TssD	Hcp	Atu4345	+	VC1415/Hcp1VCA0017/Hcp2	++	EvpC	+
		Atu4346	−	NA	NA	NA	NA
		Atu4347	−	NA	NA	NA	NA
TssI	VgrG	Atu4348	+	VC1416/VgrG-1VCA0018/VgrG-2 VCA1023/VgrG-3	++−	EvpI	+
		Atu4349	−	NA	NA	NA	NA
		Atu4350	−	NA	NA	NA	NA
		Atu4351	NA	NA	NA	NA	NA
		Atu4352	−	VCA0105	NA	EvpJ	−
TssI	VgrG	Atu3642	+	VC1416/VgrG-1VCA0018/VgrG-2VCA1023/VgrG-3	++−	EvpI	+
		NA	NA	VCA0113/SciN homolog	+	EvpL/SciN/homolog	+
		Atu1446	NA	VCA0117/VasH	+	YagV	NA
		NA	NA	VCA0118	−	NA	NA
		NA	NA	VCA0121	−	NA	NA
		NA	NA	VCA0122	−	NA	NA
		NA	NA	NA	NA	EvpD	−
		NA	NA	NA	NA	EvpP	−

(+) : Essential for Hcp secretion; (−) : Non-essential for Hcp secretion; (NA) : Not available.

a This study and [37], [44].

b [2], [11], [19], [36].

c [12].

The ability for Hcp secretion could be restored in most mutants with expression of the corresponding gene driven by constitutively expressed *lacZ* promoter on the plasmid pRL662 (Figure S1A in File S1). Δ*fha*, Δ*atu4341* (Δ*tssC_41_*), and Δ*atu4346* remained deficient in Hcp secretion with this *trans* complemention (Figure S1A in File S1). However, Hcp secretion could be restored to parental levels in Δ*fha* and Δ*tssC_41_* when the mutation was converted back to the wild type by gene replacement on the chromosome (Figure S1B in File S1), which confirms the requirement of these proteins in Hcp secretion. In contrast, Δ*atu4346* remained deficient in Hcp secretion even when Atu4346 expression was restored by gene replacement (data not shown). This result suggested that the defect of Hcp secretion in the Δ*atu4346* is caused by other mutation(s) yet to be identified rather than by the deletion of *atu4346.* Because the adjacent gene *atu4347* is dispensable for Hcp secretion and encodes a secreted exoprotein discovered in this study (see below), we generated the Δ*atu4346* Δ*atu4347* double deletion mutant to determine the requirement of *atu4346* in Hcp secretion. This mutant remains proficient in Hcp secretion (Figure 2A), which indicates that *atu4346* is not required to mediate Hcp secretion.

Therefore, we concluded that the *A. tumefaciens* T6SS machinery comprises at least 14 proteins, including 11 encoded from the *imp* operon and 3 encoded from the *hcp* operon (Figure 1A). Because of the functional redundancy of *vgrG-1* and *vgrG-2* in mediating Hcp secretion (Figs. 2A and Figure S1 in File S1), we consider VgrG-1 and VgrG-2 together as one T6SS component. While not being an essential component for Hcp secretion, the putative serine/threonine kinase PpkA also quantitatively regulates Hcp secretion, with evidence of reduced Hcp secretion levels in Δ*atu4330* (Δ*ppkA*) (Figure 2A). In contrast, deletion of *atu4339*, encoding a TagJ homolog [3], *atu4331* (*pppA*), and the non-conserved genes (*atu4346, atu4347*, *atu4349*, *atu4350*, *atu4352*) in the *hcp* operon does not significantly affect Hcp secretion under this growth condition (Figure 2A).

### Atu4347 is a Novel T6SS-secreted Exoprotein

Hcp, the hallmark of a functional T6SS, is the only T6SS-secreted protein identified by secretome analysis of *A. tumefaciens* [44]. Although the *imp* operon encodes well-conserved proteins mostly required for Hcp secretion, the *hcp* operon encodes the known or putative secreted exoproteins Hcp and VgrG-1 and several non-conserved proteins, of which most, if not all, are dispensable for Hcp secretion (Figures 1A and 2A). Therefore, these proteins may have accessory roles in the T6S machinery or are secreted T6S effectors. We noted that Atu4347, an *hcp* operon-encoded protein dispensable for Hcp secretion (Figure 2A), accumulates at higher levels in Δ*imp* (Figure 1C) than in the wild type. Because Hcp also accumulated to slightly higher levels when its secretion was blocked (Figure 2A) [44], we then tested whether Atu4347 is secreted to an extracellular milieu. Indeed, Atu4347 was detected in the culture medium of C58, even though its secreted level was less than that for Hcp (Figure 2B). In contrast, we found no evidence of Atu4349 or VgrGs secreted into culture under this growth condition (Figure 2B). Atu4347 secretion was also blocked in Δ*imp* and Δ*tssM*, where its secretion was restored to the wild-type level by *trans* complementation (Figure 2C). Thus, we identified Atu4347 as a novel T6SS-secreted protein.

### Hcp, Atu4347, and VgrGs are Exposed on the Bacterial Cell Surface

**Figure 3:**
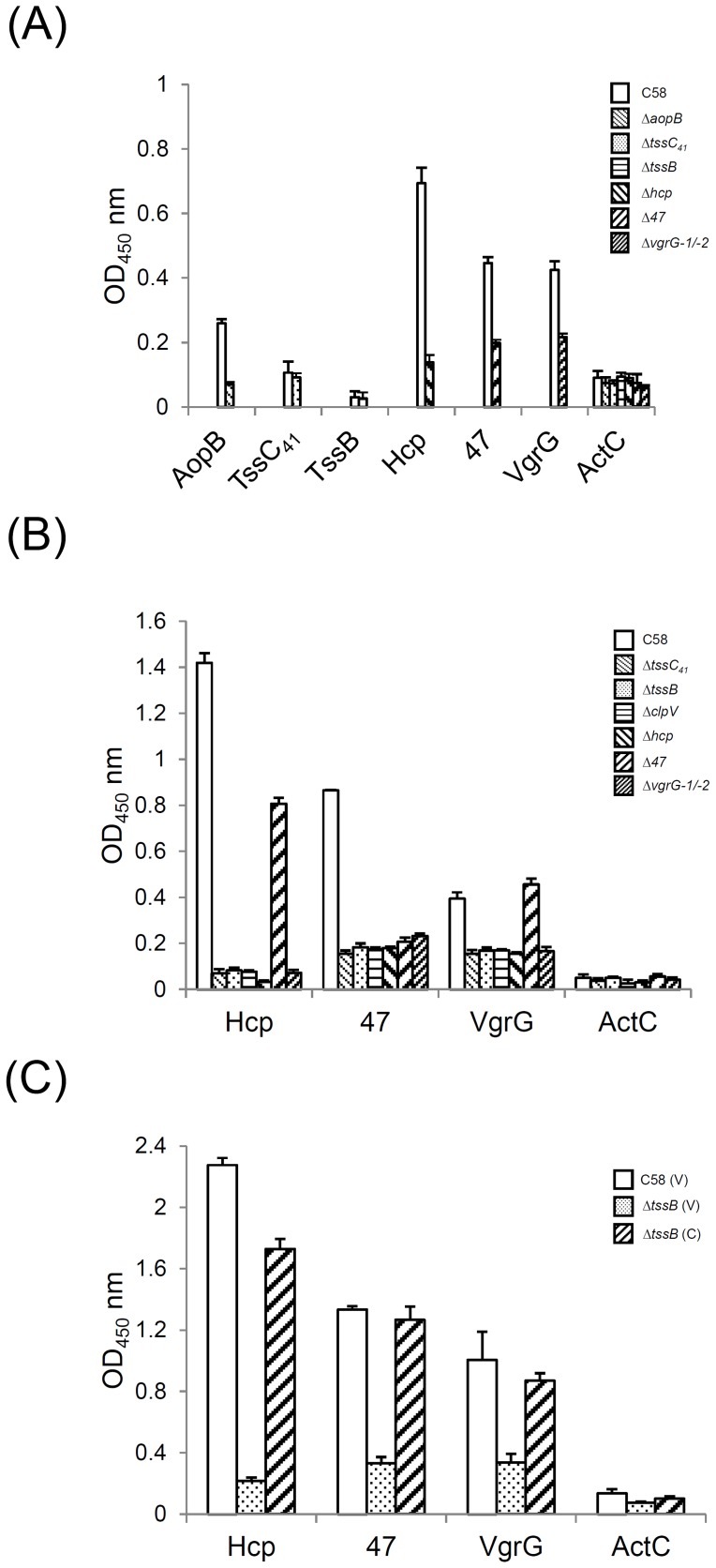
Detection of Hcp, VgrGs, and Atu4347 proteins on the bacterial cell surface by whole-cell ELISA. (**A**) Hcp, VgrGs, and Atu4347 are localized on bacterial surface. (**B**) T6SS-dependent surface localization of Hcp, VgrGs, and Atu4347. (**C**) Complementation test. *A. tumefaciens* wild-type C58, Δ*aopB*, Δ*tssC_41_*, Δ*tssB*, Δ*clpV*, Δ*hcp*, Δ*atu4347*(Δ*47*), and Δ*vgrG-1/−2* mutants or wild-type C58 and Δ*tssB* strains harboring the vector pRL662 (V) or complemented plasmid (C) grown in AB-MES (pH 5.5) for 6 h at 25°C were collected, and intact cells were used for ELISA with various antibodies. AopB, a surface protein, was used as a positive control [47], and ActC, a periplasmic protein [46], was a negative control. The strains used and proteins analyzed are indicated on the right and below, respectively. The Y-axis indicates the OD_450_ value representing the signal intensity of reaction to specific antibody. Data are mean±SD of triplicate samples.

The experimental evidence for the resemblance of T6SS to the contractile phage tail-like structure was first supported by the ability of the Hcp hexamer stacking into a tube *in vitro* when the cysteine was introduced to stabilize the ring-ring interface [17] and recently by the microscopy observation of both extended and contracted TssB–TssC tubule conformation in the cytoplasm of *V. cholerae* [22], [23]. However, whether this phage tail-like structure is assembled or penetrates across double membranes to the bacterial surface remains unclear. Thus, we attempted to detect the surface localization of the tube components Hcp and VgrGs and newly identified exoprotein Atu4347 by ELISA of intact cells. To test the suitability of this method in detecting surface protein, we used a known surface protein, AopB, an OM protein localized on the surface of *A. tumefaciens* [47], and a non-secreted periplasmic protein, ActC [37], [46], as positive and negative controls, respectively. The AopB surface signal was significantly higher on wild-type *A. tumefaciens* C58 than Δ*aopB* (Figure 3A). In contrast, signals for ActC remained at the same low background levels on all analyzed strains including Δ*actCBA* (Figure 3A and Figure S2A in File S1). Importantly, the background signal for ActC was not due to the lack of recognition of the ActC native protein by the antibody because we detected approximately three-fold higher signals when C58 cells were treated with lysozyme to expose the periplasmic proteins for antibody recognition (Figure S2A in File S1).

With the validation of this method, we then determined whether Hcp, VgrGs, and Atu4347 could be detected on the bacterial surface. We identified specific signals for Hcp, VgrGs, and Atu4347 on the cell surface by their significantly higher signals from wild-type C58 than the respective mutants (Figure 3A). In contrast, we did not detect Atu4341 (TssC_41_) or Atu4342 (TssB), two essential components with sequence homology to sheath components TssC (VipB) and TssB (VipA), respectively, because only background signals were detected from both C58 and mutants (Figure 3A). Importantly, we detected surface signals for Hcp, VgrGs, and Atu4347 only in the secretion-active strains (wild type and Δ*atu4347*) and not all analyzed secretion-deficient mutants (Δ*tssB*, Δ*tssC_41_*, Δ*clpV,* Δ*hcp*, and Δ*vgrG-1/−2*) (Figure 3B). The deficiency of their surface localization in the secretion-deficient state could be restored by complementation because the surface signals could return to the wild-type level when Atu4342 (TssB) was expressed in the Δ*atu4342* (Δ*tssB*) mutant (Figure 3C). These data strongly suggest that the translocation of T6SS exoproteins across bacterial membranes to the surface might be a prerequisite step before their secretion to the environmental milieu or host cells. Alternatively, the secreted proteins detected in the medium may derive from the yet-unidentified needle-like surface structure via physical vibration during growth.

### Interaction Studies of Hcp, VgrG, and Atu4347

**Figure 4:**
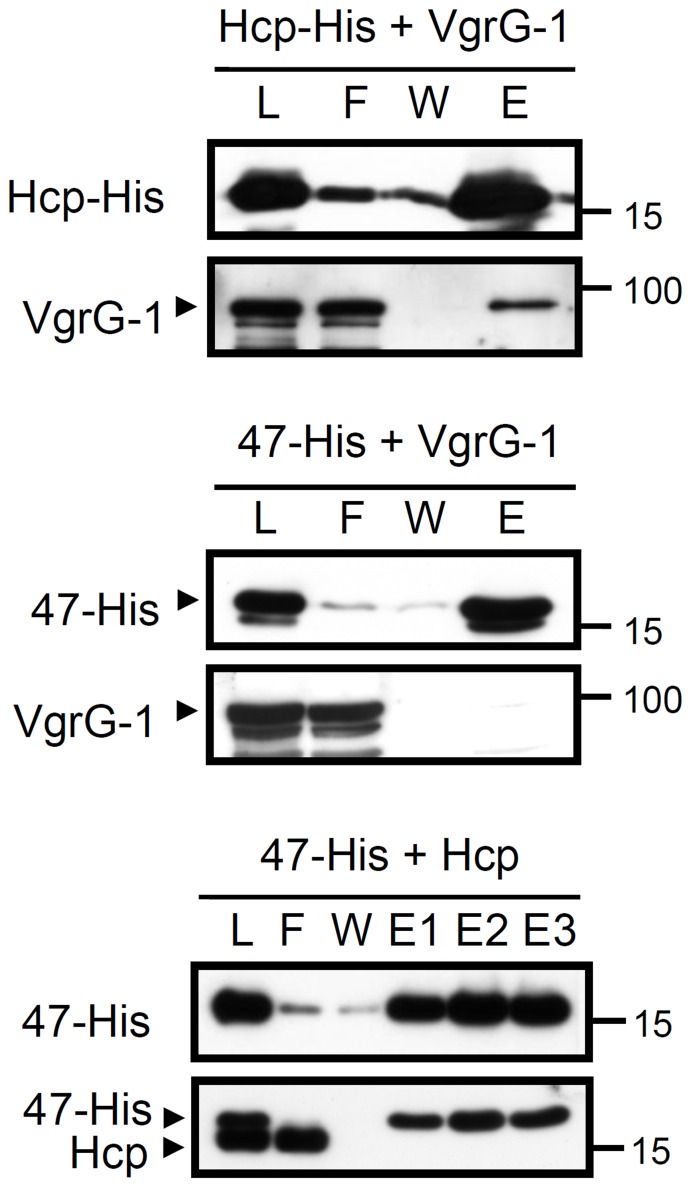
Protein–protein interaction of Hcp, VgrG, and Atu4347 in *E.* *coli*. Co-purification of VgrG-1 (pTrc-VgrG-1) with Hcp-His (pET22b-Hcp-His), Atu4347-His (pET22b-Atu4347-His), or Hcp (pTrc-Hcp) with Atu4347-His (pET22b-Atu4347-His) from *E. coli* BL21 (DE3). Proteins were induced by IPTG and the soluble protein extracts were passed through Ni-NTA His binding resins to purify His-tagged proteins and their interacting proteins. The fractions of load (L), flow-through (F), wash (W), and elution (E) were analyzed by western blot analysis with specific antibodies of Hcp, VgrG-1, or Atu4347. The proteins analyzed and sizes of molecular weight standards are indicated on the left and right, respectively, and with arrows when necessary. Hcp antibody could recognize both Hcp and His-tag fusion protein such as Atu4347-His as indicated.

The identification of surface-localized Hcp, VgrG, and Atu4347 prompted us to investigate whether these three exoproteins interact with each other. To address this question, we co-expressed them in pairs in *E. coli* and discovered the direct interaction of Hcp and VgrG-1 (Figure 4). However, Atu4347 did not interact directly with Hcp or VgrG-1 because Hcp or VgrG-1 could not be co-purified with Atu4347–His by Ni-resin (Figure 4). The same negative results were obtained when Atu4347 was co-expressed with Hcp–His or VgrG-1–His (data not shown). Thus, we provide evidence for a specific interaction between Hcp and VgrG-1.

### Atu4341 (TssC_41_) and Atu4342 (TssB) Stabilize Each Other and form a Complex *in vivo*

**Figure 5:**
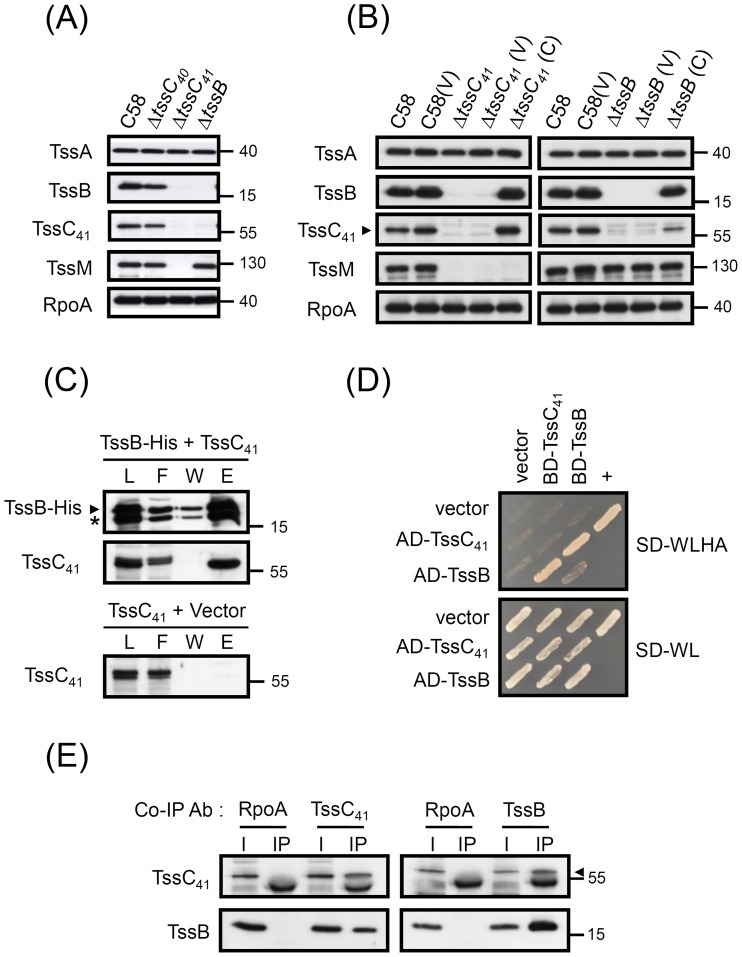
TssC_41_ and TssB stabilize each other and form a binary complex *in vivo*. (**A**) Western blot analysis of protein accumulation in *A. tumefaciens* wild-type C58 and various mutants. (**B**) Complementation test for TssC_41_ and TssB protein accumulation. Total proteins isolated from wild-type C58 or various *A. tumefaciens* strains alone, containing vector pRL662 (V) or complemented plasmid (C) were resolved by 12% Glycine-SDS-PAGE and analyzed by western blot analysis with specific antibodies. RpoA was used as an internal control. The proteins analyzed and sizes of molecular weight standards are indicated on the left and right, respectively, and with arrows when necessary. (**C**) Co-purification of TssC_41_ (pTrc-TssC_41_) with TssB-His (pET22b-TssB-His) or vector (pET22b) from *E. coli* BL21 (DE3). Proteins were induced by IPTG and the soluble protein extracts were passed through Ni-NTA His binding resins to purify His-tagged proteins and their interacting proteins. The fractions of load (L), flow-through (F), wash (W), and elution (E) were analyzed by western blot analysis with specific antibodies for TssB or TssC. Two TssB-specific protein bands are detected when expressed in *E. coli*, which suggests that a truncated TssB (*) may be formed by proteolysis due to the overexpression in *E. coli.* The proteins analyzed and sizes of molecular weight standards are indicated on the left and right, respectively, and with arrows when necessary. (**D**) Yeast two-hybrid protein–protein interaction results with TssC_41_ and TssB. SD-WL medium (SD minimal medium lacking Trp and Leu) was used for the selection of plasmids. SD-WLHA medium (SD minimal medium lacking Trp, Leu, His, and Ade) was used for the auxotrophic selection of bait and prey protein interactions. The positive interaction was determined by growth on SD-WLHA medium at 30°C for at least 2 days. The positive control (+) showing interactions of SV40 large T-antigen and murine p53 and negative control (vector) are indicated. (**E**) Co-IP of TssC_41_ and TssB in *A. tumefaciens*. Total protein extracts isolated from *A. tumefaciens* wild-type strain C58 treated with DTBP cross-linker were solubilized by buffer containing 1% SDS, then diluted into solution for IP. Co-precipitated proteins were identified by western blot analysis. Co-IP was also performed with anti-RpoA as a negative control. The proteins analyzed and sizes of molecular weight standards are indicated on the left and right, respectively, and with arrows when necessary.

To date, the interaction and/or complex formation of TssB–TssC (VipA–VipB) homologues have been demonstrated in various bacteria *in vivo* or when they are expressed in heterologous systems such as yeast or *E. coli* [19], [21], [22], [48]–[50], so the TssB–TssC complex formation may be a general feature of T6SS. However, *A. tumefaciens* TssB and TssC orthologs remain unclear because of the presence of two T6SS components with homology to TssC. Because both Atu4340 and Atu4341 have limited sequence homology to each other and are both considered TssC homologs [3], [44], we named them TssC_40_ and TssC_41_, respectively. Interestingly, the components are not functionally redundant in mediating Hcp secretion (Figure 2A) and behave differently in terms of impact on stabilizing Atu4342 (TssB) (Figure 5A). Atu4341 (TssC_41_) and Atu4342 (TssB) could accumulate at significant level in Δ*atu4340* (Δ*tssC_40_*) but were barely detectable in the absence of each other (Figure 5A, 5B). The loss of Atu4341 (TssC_41_) or Atu4342 (TssB) in the absence of each could be restored in full or in part by complementation (Figure 5B). As controls, the protein levels of Atu4343 (TssA), expressed upstream of *atu4342* (*tssB*), or non-T6SS protein, RpoA, remained the same in these strains (Figure 5A). Interestingly, TssM, a protein expressed downstream of *atu4341* (*tssC_41_*), is not detectable in Δ*atu4341* (Δ*tssC_41_*) and this deficiency is not restored by complementation (Figure 5A and 5B). Thus, deletion of *atu4341* (*tssC_41_*) may have a polar effect, which explains the failure of *trans* complementation of *atu4341* (*tssC_41_*) for Hcp secretion (Figure S1 in File S1). Nevertheless, the mutual dependence of Atu4341 (TssC_41_) and Atu4342 (TssB) may suggest their physical interaction to stabilize each other and form a complex.

To test this hypothesis, we first performed yeast two-hybrid assay and co-purification in *E. coli* and demonstrated that Atu4341 (TssC_41_) and Atu4342 (TssB) directly interact with each other to form a binary complex (Figure 5C and 5D). Furthermore, we performed co-IP to determine their interaction in *A. tumefaciens.* To avoid the non-specific protein–protein interactions occurring when proteins are released into solution after cell breakage, we used the cleavable and membrane permeable cross-linker dimethyl 3,3'-dithiobispropionimidate (DTBP) to cross-link interacting proteins before cell lysis [51] for all co-IP and pulldown assays in *A. tumefaciens.* DTBP cross-linked bacterial cells were first extracted by SDS, then diluted into TX-100–containing solution before co-precipitation. As controls, Atu4341 (TssC_41_) and Atu4342 (TssB) were not precipitated by anti-RpoA antibody (Figure 5E), and no signals of Atu4341 (TssC_41_) and Atu4342 (TssB) were detected by precipitated with their specific antibodies in the absence of each other (data not shown). Thus, Atu4341 and Atu4342 may be functional orthologs of the TssC–TssB tubule structure, directly interacting with each other to form a complex in yeast, *E. coli* and *A. tumefaciens*. Interestingly, TssB may self-interact in light of the weak interaction detected by yeast two-hybrid assay (Figure 5D).

### Interactions between TssB–TssC_41_ Complex and Exoproteins

The identity of the TssB–TssC tubule visualized in the cytoplasm of *V. cholerae* was confirmed by mass spectrometry analysis of purified structure, but only the extended form could be purified and consisted of mostly VipA (TssB) and VipB (TssC), the components of the outer sheath structure [22]. Interestingly, Hcp and VgrG, the putative internal tube components, are not identified in this purified structure [22]. If the TssB–TssC complex functions as the tail sheath to directly push the Hcp tube across bacterial membranes from interior cells by contraction, TssB–TssC should interact with Hcp directly during the Hcp secretion/assembly. Thus, we aimed to identify whether TssC_41_ and TssB interact with the putative tube components Hcp and VgrG as well as the newly identified exoprotein Atu4347.

**Figure 6:**
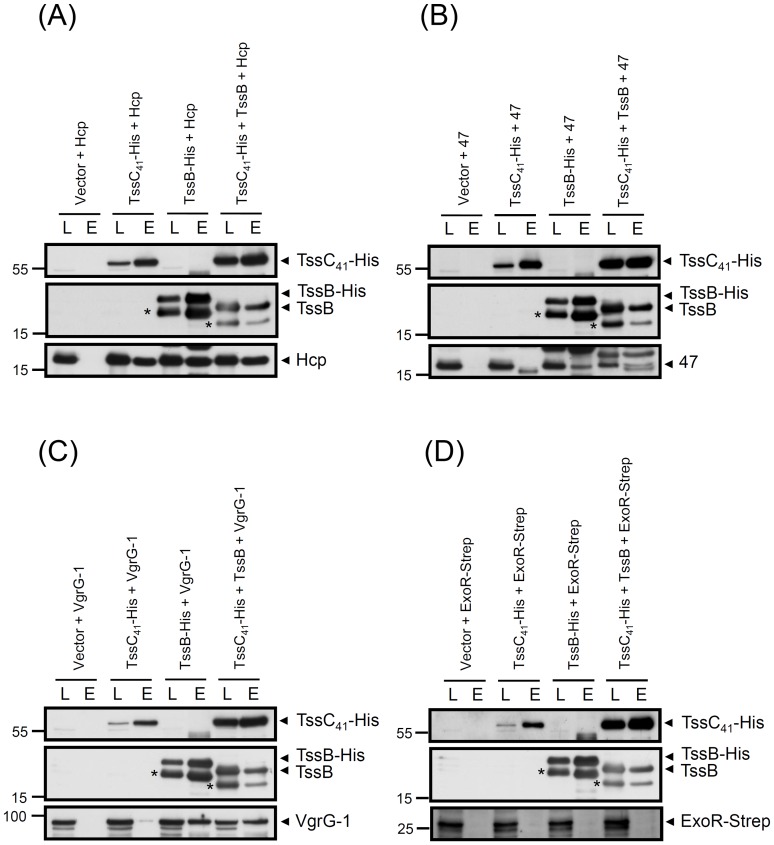
TssC_41_–TssB complex interacts directly with T6SS exoproteins in ***E.*** **coli**** **.** **** (**A**) Co-purification of Hcp (pTrc-Hcp) with vector (pET22b only), TssC_41_-His (pET22b-TssC_41_-His), TssB-His (pET22b-TssB-His), or TssB plus TssC_41_-His (pET22b-TssB-TssC_41_-His) from *E. coli* BL21 (DE3). (**B**) Co-purification of Atu4347 (pTrc-Atu4347) with vector (pET22b only), TssC_41_-His (pET22b-TssC_41_-His), TssB-His (pET22b-TssB-His), or TssB plus TssC_41_-His (pET22b-TssB-TssC_41_-His) from *E. coli* BL21 (DE3). (**C**) Co-purification of VgrG-1 (pTrc-VgrG-1) with vector (pET22b only), TssC_41_-His (pET22b-TssC_41_-His), TssB-His (pET22b-TssB-His), or TssB plus TssC_41_-His (pET22b-TssB-TssC_41_-His) from *E. coli* BL21 (DE3). (**D**) Co-purification of ExoR-Strep (pTrc-ExoR-Strep) with Vector (pET22b only), TssC_41_-His (pET22b-TssC_41_-His), TssB-His (pET22b-TssB-His), or TssB plus TssC_41_-His (pET22b-TssB-TssC_41_-His) from *E. coli* BL21 (DE3). Proteins were induced by IPTG and the soluble protein extracts were passed through Ni-NTA His binding resins to purify His-tagged proteins and their interacting proteins. The fractions of load (L), and elution (E) were analyzed by western blot analysis with specific antibodies for Hcp, TssB, TssC, or Strep epitope for ExoR-Strep. Two TssB-specific protein bands are detected when expressed in *E. coli*, which suggests that a truncated TssB (*) may be formed by proteolysis due to the overexpression in *E. coli.* The proteins analyzed and sizes of molecular weight standards are indicated on the right and left, respectively, and with arrows when necessary.

By co-purification in *E. coli*, we discovered that Hcp, VgrG-1, and Atu4347 each could interact with the TssB–TssC_41_ complex when TssB and TssC_41_–His were co-expressed in pET22b(+) (Figure 6). To further explore whether TssC_41_ or TssB interacts directly with the three exoproteins, we co-expressed TssC_41_–His or TssB–His with each of Hcp, Atu4347, and VgrG-1 to examine their direct interaction. Hcp, Atu4347, and VgrG-1 could be co-purified with TssB-His, but only Hcp but neither VgrG-1 nor Atu4347 could interact with TssC_41_ (Figure 6). The interaction between TssB and each exoprotein is specific because co-expressed control protein (ExoR-Strep) could not co-purify with TssC_41_ or TssB (Figure 6D).

The positive results from co-purification in *E. coli* then led us to further perform co-IP and pulldown assays to determine their interactions. Before the interaction studies, we first used biochemical fractionation followed by western blot analysis to investigate whether the putative sheath components and exoproteins are localized in the same subcellular compartments. All the putative sheath (TssC_41_ and TssB) and tube (Hcp and VgrG) components as well as the newly identified Atu4347 exoprotein were mainly localized in the soluble cytoplasmic fraction but also present at lesser amounts in the insoluble fraction (Figure S3 in File S1). Notably, Hcp was also present at low levels in periplasm, an observation consistent with detection of periplasmic Hcp in other systems [15], [31], [52], [53].

**Figure 7:**
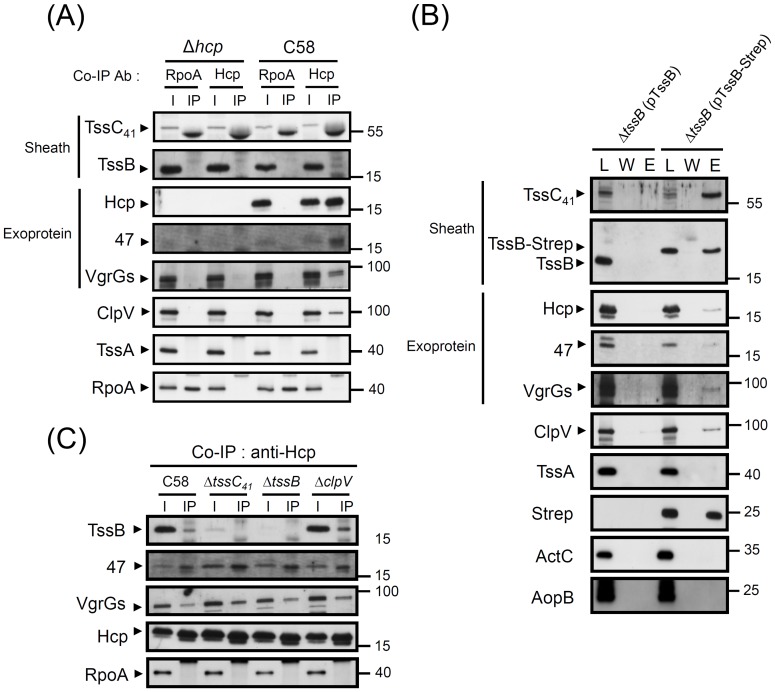
Interactions between sheath components and exoproteins in *A.* *tumefaciens*. (**A**) Co-IP of Hcp with other exoproteins and TssC_41_/TssB complex from *A. tumefaciens* wild-type strain C58 and Δ*hcp*. Total protein extracts isolated from *A. tumefaciens* cells treated with DTBP cross-linker were solubilized by buffer containing 1% SDS, then diluted into solution for IP. Co-IP was also performed with anti-RpoA as a negative control to confirm the co-IP specificity to Hcp. The proteins analyzed and sizes of molecular weight standards are indicated on the left and right, respectively, and with arrows when necessary. TssC_41_ and TssB are indicated as Sheath, and Hcp, Atu4347 and VgrG are indicated as Exoprotein. (**B**) Pulldown assay by TssB-Strep in *A. tumefaciens.* Total proteins were extracted from DTBP cross-linked bacterial cells of the Δ*tssB* mutant harboring plasmid expressing TssB or TssB tagged with Strep (TssB-Strep). The detergent solubilized proteins were used to pull down TssB-Strep and its interacting proteins. The fractions of load (L), wash (W), and elution (E) were examined by western blot analysis. The proteins analyzed and sizes of molecular weight standards are indicated on the left and right, respectively, and with arrows when necessary. TssC_41_ and TssB are indicated as Sheath, and Hcp, Atu4347 and VgrG are indicated as Exoprotein. (**C**) Co-IP of Hcp with other exoproteins from *A. tumefaciens* wild-type strain C58, Δ*tssC_41_*, Δ*tssB*, and Δ*clpV*. Total protein extracts isolated from *A. tumefaciens* cells treated with DTBP cross-linker were solubilized by buffer containing 1% SDS, then diluted into solution for IP. The proteins analyzed and sizes of molecular weight standards are indicated on the left and right, respectively, and with arrows when necessary.

In considering the possible low abundance and weak interactions among the TssB–TssC_41_ complex and exoproteins, DTBP cross-linked bacterial cells were used to extract detergent-solubilized proteins from total cell lysates for co-precipitation. By co-IP with anti-Hcp, we were able to co-IP significant amounts of VgrGs and Atu4347 as well as TssB and ClpV, although at low levels (Figure 7A). Conversely, use of Strep-tagged TssB, with full function in mediating Hcp secretion (Figure S4 in File S1), as a bait for pulldown, revealed its strong interaction with TssC_41_ and weak interaction with ClpV and the three exoproteins (Hcp, VgrG, and Atu4347) (Figure 7B). As a control, use of the Δ*tssB* mutant expressing TssB without a Strep tag or Δ*hcp* mutant revealed none of these proteins in the elution fraction. Furthermore, another T6SS protein, TssA, and non-T6SS proteins such as RpoA, ActC, or AopB were not present in co-precipitates, which suggests the specific interactions of identified proteins. Interestingly, the interactions among the three exoproteins and with TssB were not affected in Δ*clpV* (Figure 7C), which is consistent with the role of ClpV in TssB–TssC sheath disassembly rather than assembly [19], [22], [23]. That the exoprotein complex formation was not affected in Δ*tssC_41_* and Δ*tssB* also suggests that the interactions are independent of the formation of a sheath complex. In summary, Hcp directly interacts with VgrG and may assemble into a tube structure to direct Atu4347 exoprotein secretion. The Hcp–VgrG–Atu4347 complex forms before their interactions with TssB and/or TssC, which interact to form a complex and may assemble around the Hcp–VgrG tube to propel this potential puncturing device across double membranes for effector secretion.

## Discussion

T6SS is a highly conserved protein secretion system that has evolved to contribute diverse biological functions. Since the proposal of the T6SS multiprotein channel resembling a phage tail structure, accumulating research has provided many exciting new insights into its structure and secretion mechanisms. However, the molecular details underlying how the phage tail-like components interact and assemble into a functional secretion apparatus remain largely unknown. In this study, we used a systematic approach to comprehensively characterize the machinery and secreted components of *A. tumefaciens* T6SS and showed that T6SS forms one or more subcomplexes among the phage tail-like components.

By determining the essentiality of each gene encoded in the T6SS gene cluster in mediating Hcp secretion into culture medium, we identified 14 T6SS proteins, including Hcp and VgrG, as the machinery components. All 14 T6SS machinery components identified in *A. tumefaciens* are conserved proteins, similar to what was previously reported for *E. tarda* [12] and *V. cholerae* [36], but they do not share all essential components (Table 1 and Table S3 in File S1). Fha is conserved in *A. tumefaciens* and *V. cholerae* but is absent in *E. tarda*. However, both *V. cholerae* and *E. tarda* contain the homologs to OM lipoprotein TssJ (SciN) of enteroaggregative *Escherichia coli* (EAEC), which is required for Hcp secretion [12], [36], [40], but we identified no TssJ homolog in *A. tumefaciens* (Table 1). The dispensability of TagJ for Hcp secretion in *A. tumefaciens* is also consistent with its absence in *V. cholerae* and *E. tarda.* However, recent identification of the TagJ–TssB complex in *P. aeruginosa* raises an interesting notion that TagJ may modulate TssB function [54]. In *A. tumefaciens,* VgrG-1 and VgrG-2 can functionally complement each other for Hcp secretion, similar to the redundant role of VgrG1a and VgrG1c in *P. aeruginosa* [13]. In contrast, VgrG-1 and VgrG-2 are each required for Hcp secretion from *V. cholera* [11]. Interestingly, secretion of VgrG has been thought to depend on T6SS and Hcp, but T6SS-independent secretion of VgrG was also identified [13]. The functional variation of VgrG proteins in different bacterial species suggests their distinct roles and specific functions in T6SS.

The *hcp* operon contains nine genes encoding three conserved components (ClpV, Hcp, VgrG-1) and six non-conserved proteins, including the newly identified T6SS-secreted exoprotein Atu4347, which are dispensable for Hcp secretion. Interestingly, Atu4347 and several non-conserved proteins dispensable for Hcp secretion are predicted to be non-classical secreted proteins [55] (Table S3 in File S1) and therefore may function as T6SS-secreted effectors contributing to specific biological functions. Because we detected no virulence phenotype in the mutant with deletion of the entire *imp* operon or *tssM* and only a minor virulence phenotype in Δ*hcp,* T6SS may not play a critical role in the virulence of *A. tumefaciens* [44]. Consistent with this conclusion, with tumor assays of tomato stems, we observed no virulence phenotype in Δ*atu4347* as compared to the wild type (data not shown). Interestingly, Atu4347 and Atu4346 were recently reported to be orthologs of the anti-bacterial toxin secreted small protein and the cognate partner resistance associated protein, a novel toxin-immunity system contributing anti-bacterial activity in *Serratia marcescens* [32]. Indeed, we identified the two conserved motifs predicted to mediate peptidoglycan amide bond hydrolysis by Russell et al. (2012) [56] in Atu4347 (data not shown), which supports a potential function of *A. tumefaciens* T6SS in bacterial killing. Our evidence that Atu4347 is a secreted exoprotein and both Atu4346 and Atu4347 are dispensable for Hcp secretion is consistent with their potential functions as a toxin-immunity pair. Our inability to obtain the deletion mutant for *atu4351* may also imply its role as another immunity protein, which is consistent with the observation of normally more than one toxin-immunity pair in each T6SS system. We are currently investigating whether the *A. tumefaciens* T6SS has anti-bacterial activity and if so, the molecular mechanisms underlying this phenotype.

Biochemical and structural analysis revealed that the VgrG forms a trimeric complex similar to the T4 phage tail spike (gp5)_3_–(gp27)_3_ complex [11], [16]. The crystal structure of the N-terminal fragment of VgrG from uropathogenic *E. coli* CFT073 revealed two domains with similar structures to the gp27 tube domain that participates in binding the entire (gp5)_3_–(gp27)_3_ complex to the tail tube [16]. Importantly, the Hcp-family protein also revealed significant homology to the tail tube protein gp19, and the *P. aeruginosa* Hcp1 is the structural homolog of gp5; its hexamer can be structurally superimposed on a trimeric pseudohexamer formed by the tube domains of gp27 [16], [18]. Taken together with the mutual requirement of Hcp and VgrG for their secretion, VgrG is generally believed to directly interact with Hcp by sitting on top of the Hcp tube, but experimental evidence is lacking. Here, we provide compelling evidence for the direct binding of Hcp and VgrG both in *A. tumefaciens* and when expressed in *E. coli.* The detection of surface-localized Hcp, VgrG, and Atu4347, together with their interactions, further support the putative role of the Hcp–VgrG tube protruding from the bacterial surface for secretion of exoproteins (such as Atu4347). In contrast to the T6SS-dependent surface localization of Hcp, VgrG, and Atu4347, that of the acid-inducible OM protein AopB [47] is independent of T6SS because levels of surface signals were similar on both wild-type C58 and secretion-deficient mutants (Δ*tssC_41_* and Δ*tssB*) (Figure S2B in File S1). Interestingly, the specific surface-localized Hcp signal was slightly but significantly lower in Δ*aopB* than in wild-type C58 (Figure S2B in File S1), which implies an accessory role of AopB in Hcp exposure to the bacterial cell surface. Because the *A. tumefaciens* T6SS is also activated by acidity [45] and does not encode the TssJ (SciN) OM lipoprotein homolog [40] (Table 1), we tested whether AopB may replace the role of TssJ in connecting the IM protein complex to mediate Hcp secretion in *A. tumefaciens*. However, Hcp was expressed and secreted into the culture medium at similar levels from wild-type C58 and Δ*aopB* (Figure S2C in File S1). Thus, AopB does not play an essential role like TssJ in mediating Hcp secretion. Whether it may quantitatively modulate Hcp secretion to the bacterial surface and/or extracellular milieu from *A*. *tumefaciens* awaits future investigation.

The detection of surface-localized VgrG but not the secreted form released into the culture medium may be due to a low level or instability of VgrG in the culture medium. In contrast, the TssB–TssC_41_ complex, a putative sheath structure engulfing the Hcp–VgrG tube, was neither secreted nor detected on the bacterial surface. Furthermore, we detected no specific signals for TssB and TssC_41_ when wild-type C58 cells were treated with lysozyme to expose the periplasmic proteins for antibody recognition (Figure S2A in File S1). This result is consistent with biochemical fractionation data revealing TssB and TssC_41_ localized in both the soluble and insoluble cytoplasmic fraction but not the periplasmic fraction (Figure S3 in File S1). Surface localization of Hcp was detected in *F. novicida* via surface biotinylation analysis, with IglC (Hcp homolog) detected on the bacterial surface in a T6SS-dependent manner [57]. However, this study also detected the surface localization of IglA (TssB) and IglB (TssC) from the wild type and *tssM* (*icmF*) or *tssL* (*dotU*) mutants. To this end, we cannot exclude that the background signal for TssB and TssC_41_ from our whole-cell ELISA may be caused by the inability of the antibodies to recognize the native TssB–TssC_41_ structure. Therefore, the Hcp–VgrG tube structure is likely exposed to the outside of the bacterial cell, but whether the outer sheath structure may also protrude across the OM requires further investigation.

Our co-precipitation studies of *A. tumefaciens* and heterologous *E. coli* provided evidence for the interactions among three exoproteins, Hcp, VgrG, and Atu4347, which also interact directly with the TssB–TssC_41_ complex. In considering the postulated role of the Hcp-VgrG tube structure responsible for exoprotein secretion, we were surprised to find no direct interaction between Atu4347 and Hcp–VgrG when expressed in *E. coli*. However, Hcp and Atu4347 could be co-precipitated by each other at an abundant level in *A. tumefaciens* (Figure 7), which suggests that the physical interaction of these 2 proteins may occur only in *A. tumefaciens*, where other T6SS components are present for assembly of the Hcp tube. However, neither the TssB–TssC sheath component nor ClpV are required for the exoprotein complex formation. In the contractile phage T4, the (gp5)_3_–(gp27)_3_ spike complex interacts with wedge proteins to form a base plate, which functions as an assembly nucleus for polymerization of the gp19 tube that is then wrapped by the outer sheath into the extended structure [58]. Our data also suggest that the formation of the Hcp–VgrG complex and its association with non-structural secreted protein occur before or independent of the assembly or disassembly of sheath complex. However, whether this exoprotein complex represents the polymerized tube remains unknown. The interaction between Hcp and another non-structure exoprotein, EvpP, in *E. tarda* [12] further supports the role of the Hcp tube as a conduit or guide for exoprotein secretion. The internal diameter (40 ,) of the Hcp hexamer ring [14], [15], [17] is sufficient to serve as a conduit to deliver folded or partially folded exoproteins. However, the future challenge is visualizing the spatial and temporal Hcp tube association with exoproteins to determine the role of the Hcp tube in exoprotein secretion.

The TssB–TssC tubular structure is a cogwheel-like structure with a 100 , central pore [19], which is sufficient for engulfing the Hcp hexamer (∼85 ,) [14], [15]. Our data showing Hcp, VgrG, and Atu4347 interacting with the TssB–TssC_41_ complex through their direct binding to TssB indeed support this phage tube/sheath structure model. However, as compared with the abundance of Atu4347 co-precipitating with Hcp, TssB and TssC_41_ levels were low in interacting with Hcp in the same experiment. For TssB pulldown, abundant TssC_41_ but only low levels of three exoproteins were co-purified with TssB. These data suggest strong or stable interactions among the three exoproteins and between TssB and TssC_41_ but only weak interaction of TssB–TssC_41_ with the exoproteins. The strong interaction between TssB and TssC_41_ is also observed from *E. coli* co-purification because the co-purified proteins could be detected by Coomassie blue staining (Figure S5 in File S1). In contrast, Hcp, VgrG-1, and Atu4347 co-purified with TssB-His and/or the TssC_41_-His–TssB complex was specifically detected by only western blot analysis but not Coomassie blue staining, which suggests the weaker interactions between the sheath complex and exoproteins. This weak interaction could also explain the previous negative results from interaction studies between the gp19 tube and gp18 sheath of phage T4 [58], and *V. cholerae* Hcp and VgrG-2 with TssB (VipA) or TssC (VipB) by pulldown assay [19]. Thus, the Hcp–VgrG tube may only weakly interact with the TssB–TssC outer sheath in a dynamic manner, which is regulated by the extension and contraction of the outer sheath during the secretion process. Hcp and VgrG proteins not detected in the purified contracted TssB–TssC (VipA–VipB) sheath structure [22] indeed supports the dynamic or weak interaction between the outer sheath and inner tube.

The co-precipitation of ClpV and other tail components with Hcp antibody prompted us to determine whether ClpV interacts directly with Hcp in addition to a known interaction with the TssB–TssC complex [19], [20]. However, we found no evidence for interactions between ClpV with exoproteins, including Hcp, VgrG-1, and Atu4347, by co-purification in *E. coli* (data not shown). Because only VipA (TssB) and VipB (TssC) but not Hcp could be co-precipitated with ClpV of *V. cholera* by *in vitro* pulldown assay [19], the co-IP of ClpV with Hcp from *A. tumefaciens* is likely due to its interaction with TssB–TssC or proteins yet to be identified. ClpV also specifically binds to the contracted TssB–TssC sheath for its disassembly and cycling, and its ATPase activity is required for the dynamic cycles of these structures [23], [33]. The temporal and spatial regulation of the assembly and disassembly of the TssB–TssC outer sheath and Hcp tube remain unknown. Future work to investigate the protein–protein interactions and complex formation in specific subcellular location and each of the T6SS mutants may allow us to dissect the order and hierarchy in the assembly of this phage tail-like structure.

## Materials and Methods

### Bacterial Strains and Growth Conditions

Strains, plasmids, and primer sequences used in this study are in Tables S1 and S2 in File S1. The growth conditions and Hcp secretion assays were as described previously [37], [44]. The plasmids were maintained by the addition of 50 µg/ml gentamycin (Gm) for *A. tumefaciens* and 100 µg/ml ampicillin (Ap), 100 µg/ml spectinomycin (Sp), 20 µg/ml kanamycin (Km), and 50 µg/ml Gm for *E. coli.*

### Plasmid Construction and Generation of in-frame Deletion Mutants

All in-frame deletion mutants were generated in *A. tumefaciens* C58 via double crossover using the suicide plasmid pJQ200KS [59] as described [37], [44]. The detailed procedures for the construction of plasmids and mutant strains are described in Information S1 in File S1.

### Antibody Production

The expression vector pET22b(+) was used to overexpress proteins driven by the T7 promoter by isopropyl-beta-D-thiogalactoside (IPTG) induction in *E. coli* BL21(DE3). The methods for protein expression and purification via Ni^2+^-NTA column (Novagen) were as described [44]. Each purified protein was separated by SDS-PAGE, and the protein band was cut out for polyclonal antibody production in rabbits.

### Total RNA Extraction and RT-PCR

Total RNA was isolated from *A. tumefaciens* strains grown in AB-MES (pH 5.5) for 6 h at 25°C by use of hot phenol as described previously [60] with minor modifications. RT-PCR was performed essentially as described [61]. An amount of 25 ml of *A. tumefaciens* strain culture was rapidly cooled to 0°C on crushed ice. The cells were pelleted and resuspended in ice-cold 125 µl of solution containing 0.3 M sucrose and 10 mM sodium acetate (pH 4.5). An equal volume of solution (2% SDS, 10 mM sodium acetate) was added, and the resulting cell suspension was heated for 3 min at 70°C and extracted 3 times for 3 min at 70°C with 250 µl preheated acidic phenol (pH 4.5, Amresco). The extracted RNA was added with 2-fold volume of ice-cold 95% ethanol and stored at −70°C for 2 hr before centrifugation at 12,000 g and 15 min at 4°C. The precipitated RNA pellet was resuspended in 50 µl diethyl pyrocarbonate (DEPC)-treated water and stored at −70°C until use.

An amount of 5 mg isolated RNA was treated with 10 units of RNase-free DNase I (Roche, Basel, Switzerland) at room temperature for 2 h. The treated RNA was purified by phenol/chloroform extraction, then ethanol precipitation and resuspended in 25 µl DEPC-treated water. An amount of 1 mg DNA-free RNA was used to synthesize cDNA with SuperScrip III RNase H2 Reverse Transcriptase (Invitrogen, Carlsbad, CA) with specific 3′-primers (Table S2 in File S1). The resulting cDNA was used as the template for PCR reaction. PCR cycles were optimized to detect the amplified products before saturation (no more than 30 cycles).

### Yeast Two-hybrid Assay

The Matchmaker yeast two-hybrid system was used according to the instructions of the user manual (Clontech, Mountain View, CA). Each of the plasmid pairs were co-transformed into *Saccharomyces cerevisiae* strain AH109. The transformants were selected by their growth on synthetic dextrose (SD) minimal medium lacking tryptophan (Trp) and leucine (Leu) (SD-WL medium). The positive interaction of expressed fusion proteins was then determined by their growth on SD lacking Trp, Leu, adenine (Ade), and histidine (His) (SD-WLHA medium) at 30°C for at least 2 days.

### Cross-linking, Co-immunoprecipitation (co-IP), and Pulldown Assay in *A. tumefaciens*

The cleavable and membrane permeable cross-linker DTBP (Sigma, Inc.) was used to cross-link interacting proteins before cell lysis and co-IP as described [51] with modifications. In total, 500 ml of *A. tumefaciens* cell culture was centrifuged and washed 3 times with 12 ml phosphate buffer (20 mM sodium phosphate, pH 7.6; 20 mM sodium chloride), and resuspended in the same buffer adjusted to OD_600_ 4. DTBP was added at a final concentration of 5 mM, and the mixture was incubated at room temperature for 45 min. The reaction was stopped by adding Tris-HCl (pH 7.6) to a final concentration of 20 mM for 15 min. The cells were collected by centrifugation and washed twice with 12 ml of 50 mM Tris-HCl (pH 7.6) before co-IP or pulldown assay.

Co-IP was performed as described [62] with modifications. All incubation steps were performed with rotary shaking. The cross-linked cells were pelleted and resuspended in 4 ml TES buffer (50 mM Tris-HCl pH 6.8, 2 mM EDTA, 1% SDS) to OD_600_ 20 before incubation for 30 min at 37°C. In total, 18 ml NP1 buffer (150 mM Tris-HCl pH 8.0, 0.5 M sucrose, 10 mM EDTA) supplemented with 1.5 mg/ml lysozyme was added for incubation for 2 h on ice, then 30 min at 37°C. Triton X-100 was added to a final concentration of 4% for incubation for 20 min at room temperature. Protease inhibitor cocktail was added to the working concentration (1X) for incubation for 15 min at 37°C, then at least 3 h at 4°C. In total, 64 ml NP1 buffer was added, and insoluble material was removed by centrifugation for 15 min at 14,000×*g*, for detergent-solubilized solution. For each 2 ml of the detergent-solubilized solution, a 60-µl bed volume of Protein A-Sepharose CL4B (Pharmacia) was added for 60-min incubation, followed by centrifugation at 5,000×*g* to remove Protein A-Sepharose and non-specifically bound proteins. The supernatant was directly incubated with each antibody with optimized titers and Protein A-Sepharose overnight at 4°C. The beads were pelleted by centrifugation and washed twice with NP1 buffer supplemented with 1% Triton X-100 and once with NP1 buffer supplemented with 0.1% Triton X-100. Co-IP beads were eluted by incubation at 96°C in 100 µl 2X SDS loading buffer for 20 min before SDS-PAGE analysis.

The pulldown assay with Strep-Tag was performed according to the user manual (Novagen). For pulldown assay with TssB-Strep, the detergent-solubilized materials described for co-IP were diluted 2-fold by use of Buffer W (100 mM Tris-HCl, pH 8.0; 150 mM NaCl; 1 mM EDTA) and passed through *Strep-*Tactin resins (GE Healthcare), washed 5 times with Buffer W1 (100 mM Tris-HCl, pH 8.0; 150 mM NaCl; 1 mM EDTA), twice with Buffer W2 (100 mM Tris-HCl, pH 8.0; 150 mM NaCl; 1 mM EDTA; 0.25 mM desthiobiotin). The bound proteins were eluted with use of Buffer E (100 mM Tris-HCl, pH 8.0; 150 mM NaCl; 1 mM EDTA; 2.5 mM desthiobiotin).

### *E. coli* Co-purification

*E. coli* co-purification was performed as described [37] with modifications. Plasmids expressing proteins without any fusion (pTrC-TssC_41_, pTrc-Hcp, pTrc-Atu4347, and pTrc-VgrG-1) or with C-terminal Strep (pTrc-ExoR-Strep) or with C-terminal His (pET-TssB-His, pET-TssC_41_-His, pET-TssB-TssC_41_-His, pET-Hcp-His, and pET-Atu4347-His) are listed in Table S1 in File S1. An overnight culture of *E. coli* BL21(DE3) cells was subcultured at a 1∶50 dilution into fresh LB medium grown to OD_600_ 0.4∼0.6 at 25°C_._ IPTG was added to a final concentration of 0.5 mM and cells were incubated at 25°C for 4 hr. The harvested cells were resuspended in 10 to 15 ml lysis buffer (50 mM NaH_2_PO_4_, 300 mM NaCl, 10 mM imidazole, 1 mM PMSF, pH 8.0) and sonicated on ice. The cell lysate was centrifuged at 20,000×g for 15 min at 4°C. The soluble fraction was filtered with use of a 0.22 µm filter and loaded onto a Ni^2+^-NTA column (Novagen), washed with washing buffer (50 mM NaH_2_PO_4_, 0.3 M NaCl, 25 mM imidazole, pH 8.0), and bound proteins were eluted using elution buffer (50 mM NaH_2_PO_4_, 300 mM NaCl, 400 mM imidazole, pH 8.0) for SDS-PAGE analysis.

### Protein Secretion Assay

*A. tumefaciens* cells grown in AB-MES medium (pH 5.5) [63] at 25°C for 6 hr were used for protein secretion assays as described [37], [44]. We collected 1 and 2 ml of culture medium for concentration by *trichloroacetic acid (TCA)* precipitation for secretion analysis of Hcp and Atu4347, respectively.

### Enzyme-linked Immunosorbent Assay (ELISA) of Intact Cells

ELISA was as described [47] with minor modification. The bacterial cells were collected and washed twice with PBS buffer (pH7.4) by centrifugation at 10,000×g for 5 min, and the bacterial cell concentration was adjusted to OD_600_ 0.5. In total, 50 µl cell suspension was transferred to each well of microtiter plates (Basic Life Bioscience, Inc.) and fixed by the addition of 50 µl of 8% paraformaldehyde for 45 min at room temperature. Cells were washed twice with 200 µl PBS (pH 7.4) and blocked with 200 µl of 2% bovine serum albumin in PBS for 45 min. For lysozyme treatment, cells were incubated with 200 µl lysozyme buffer [37] (50 mM Tris-HCl, 20% sucrose, 2 mM EDTA, 0.2 mM DTT, 10 mM MgSO_4_, 2 mg/ml lysozyme, pH7.5) for 15 min before blocking. After 3 washes with PBS, cells were incubated for 45 min with specific antibodies with optimized titer in PBST/BSA (PBS, 0.02% Tween 20, 0.2% BSA). Cells were washed 3 times with 200 µl PBST (PBS, 0.05% Tween 20) for 10 min each, then incubated with 200 µl secondary antibodies (1∶20000 dilution) for 45 min. Cells were washed at least 3 times for 10 min each with 200 µl PBST before 150 µl 1-Step Ultra TMB-ELISA (PIERCE) was added to each well for incubation in the dark for 15 min. The reaction was stopped by the addition of 50 µl of 1 N H_2_SO_4_. Absorbance at 450 nm was measured by use of an ELISA reader (PowerWave XS2, Microplate Spectrophotometer, BioTek Instruments, Inc.).

### Western Blot Analysis

Western blot analysis was as described previously [63] with primary polyclonal antibodies produced in this study and those against C-TssM [37], Hcp [44], and ActC [46], monoclonal antibodies against hemagglutinin (HA) (Sigma, Inc.), or monoclonal antibodies against Strep (GE Healthcare), followed by the secondary antibody horseradish peroxidase (HRP)-conjugated goat anti-rabbit IgG (chemichem) and detected by the Western Lightning System (Perkin Elmer, Boston, MA). Chemiluminescent bands were visualized by use of X-ray film (Kodak, Rochester, NY).

## Supporting Information

File S1 **Contains: Information S1; Figure S1. Complementation analysis of identified mutant impaired in Hcp secretion.** **(A)** Complementation test of the identified mutants deficient in Hcp secretion. The wild-type C58 and various mutants alone or harboring the vector pRL662 (V) or complemented plasmid (C) were analyzed for Hcp secretion. **(B)** Hcp secretion analysis of *fha* and *tssC_41_* revertants. Total (T) and secreted (S) proteins isolated from wild-type C58 and various strains grown in AB-MES (pH 5.5) for 6 h at 25°C were separated by 12% Glycine-SDS-PAGE and examined by western blot analysis. The secreted proteins were collected from 1 ml of culture medium after removal of bacterial cells by centrifugation and were concentrated by TCA precipitation [44]. The non-secreted protein ActC was an internal control. The proteins analyzed and sizes of molecular weight standards are indicated on the left and right, respectively. **Figure S2. Whole-cell ELISA and Hcp secretion assay.** **(A)** ActC signals were significantly increased from wild-type C58 with lysozyme treatment. *A. tumefaciens* wild-type C58 and Δ*actCBA* grown in AB-MES (pH 5.5) for 6 h at 25°C were collected, and intact cells were treated with lysozyme (Lysozyme) or without lysozyme (None) and used for ELISA with various antibodies. **(B)** AopB surface localization is independent of T6SS. *A. tumefaciens* wild-type C58, Δ*aopB*, Δ*tssC_41_*, and Δ*tssB* grown in AB-MES (pH 5.5) for 6 h at 25°C were collected, and intact cells were used for ELISA with various antibodies. The strains used and proteins analyzed are indicated on the right and below, respectively. The Y-axis indicates the OD_450_ value representing the signal intensity of reaction to specific antibody. Data are mean±SD of triplicate samples. **(C)** AopB does not significantly affect secretion of Hcp. Total (T) and secreted (S) proteins isolated from wild-type C58 and two Δ*aopB* strains grown in AB-MES (pH 5.5) for 6 h at 25°C were separated by 12% Glycine-SDS-PAGE and examined by western blot analysis with specific antibodies. The secreted proteins were collected from 1 ml of culture medium after removal of bacterial cells by centrifugation and were concentrated by TCA precipitation [44]. The non-secreted protein ActC was an internal control. The proteins analyzed and sizes of molecular weight standards are indicated on the left and right, respectively. **Figure S3. Biochemical fractionation of sheath components and exoproteins.** Equal volumes of total proteins (T), periplasmic fraction (P), proteins from Spheroplast (Sp), soluble cytoplasmic fraction (S), and insoluble fraction (IS) isolated from *A. tumefaciens* wild-type C58 were separated by 10% or 12% Glycine-SDS-PAGE followed by western blot analysis. Proteins analyzed with antibodies against specific proteins are indicated on the left and sizes of molecular weight standards are indicated on the right, and with arrows when necessary. The quality of biochemical fractionation was monitored by TssM used as insoluble protein markers, ActC as a periplasmic protein marker [37], [46], and Fha as a cytoplasmic protein marker. **Figure S4. Strep-tagged TssB has full function in mediating Hcp secretion.** The Δ*tssB* mutant harboring the vector pRL662 (V) or complemented plasmid (C) or complemented plasmid with C-terminal Strep tag (C-Strep) were analyzed for Hcp secretion. Total (T) and secreted (S) proteins isolated from wild-type C58 and various strains grown in AB-MES (pH 5.5) for 6 h at 25°C were separated by 12% Glycine-SDS-PAGE and examined by western blot analysis. The non-secreted soluble protein ActC was an internal control. The proteins analyzed and sizes of molecular weight standards are indicated on the left and right, respectively. **Figure S5. Commassie blue staining of co-purified fractions from** ***E. coli*** **.** **(A)** Co-purification of Hcp (pTrc-Hcp) with Vector (pET22b only), TssC_41_-His (pET22b-TssC_41_-His), TssB-His (pET22b-TssB-His), or TssB plus TssC_41_-His (pET22b-TssB-TssC_41_-His) from *E. coli* BL21 (DE3). **(B)** Co-purification of Atu4347 (pTrc-Atu4347) with Vector (pET22b only), TssC_41_-His (pET22b-TssC_41_-His), TssB-His (pET22b-TssB-His), or TssB plus TssC_41_-His (pET22b-TssB-TssC_41_-His) from *E. coli* BL21 (DE3). **(C)** Co-purification of VgrG-1 (pTrc-VgrG-1) with Vector (pET22b only), TssC_41_-His (pET22b-TssC_41_-His), TssB-His (pET22b-TssB-His), or TssB plus TssC_41_-His (pET22b-TssB-TssC_41_-His) from *E. coli* BL21 (DE3). **(D)** Co-purification of ExoR-Strep (pTrc-ExoR-Strep) with Vector (pET22b only), TssC_41_-His (pET22b-TssC_41_-His), TssB-His (pET22b-TssB-His), or TssB plus TssC_41_-His (pET22b-TssB-TssC_41_-His) from *E. coli* BL21 (DE3). Proteins were induced by IPTG and the soluble protein extracts were passed through Ni-NTA His binding resins to purify His-tagged proteins and their interacting proteins. The fractions of load (L), and elution (E) were analyzed by western blot analysis of Hcp, VgrG-1, Atu4347, TssB, or TssC. Two TssB-specific protein bands are detected when expressed in *E. coli*, which suggests that a truncated TssB (*) may be formed by proteolysis due to the overexpression in *E. coli.* The proteins analyzed and sizes of molecular weight standards are indicated on the right and left, respectively, and with arrows when necessary. All the samples were also analyzed by SDS-PAGE followed by Coomassie blue staining; the positions of putative TssC_41_-His, TssB-His, and TssB proteins are indicated by arrows. **Table S1. Bacterial strains and plasmids. Table S2. Primers used in this study. Table S3. Characteristics of proteins encoded by the** ***imp*** **cluster.**
(PDF)
