# Supplementary material for: Systematic Dissection of the Agrobacterium Type VI Secretion System Reveals Machinery and Secreted Components for Subcomplex Formation
Source: PLoS One. 2013 Jul 5;8(7):e67647. doi: 10.1371/journal.pone.0067647 (PMC3702570; doi:10.1371/journal.pone.0067647)
Supplement: File S1 — Contains: Information S1; Figure S1. Complementation analysis of identified mutant impaired in Hcp secretion. (A) Complementation test of the identified mutants deficient in Hcp secretion. The wild-type C58 and various mutants alone or harboring the vector pRL662 (V) or complemented plasmid (C) were analyzed for Hcp secretion. (B) Hcp secretion analysis of fha and tssC41 revertants. Total (T) and secreted (S) proteins isolated from wild-type C58 and various strains grown in AB-MES (pH 5.5) for 6 h at 25°C were separated by 12% Glycine-SDS-PAGE and examined by western blot analysis. The secreted proteins were collected from 1 ml of culture medium after removal of bacterial cells by centrifugation and were concentrated by TCA precipitation [44]. The non-secreted protein ActC was an internal control. The proteins analyzed and sizes of molecular weight standards are indicated on the left and right, respectively. Figure S2. Whole-cell ELISA and Hcp secretion assay. (A) ActC signals were significantly increased from wild-type C58 with lysozyme treatment. A. tumefaciens wild-type C58 and ΔactCBA grown in AB-MES (pH 5.5) for 6 h at 25°C were collected, and intact cells were treated with lysozyme (Lysozyme) or without lysozyme (None) and used for ELISA with various antibodies. (B) AopB surface localization is independent of T6SS. A. tumefaciens wild-type C58, ΔaopB, ΔtssC41, and ΔtssB grown in AB-MES (pH 5.5) for 6 h at 25°C were collected, and intact cells were used for ELISA with various antibodies. The strains used and proteins analyzed are indicated on the right and below, respectively. The Y-axis indicates the OD450 value representing the signal intensity of reaction to specific antibody. Data are mean±SD of triplicate samples. (C) AopB does not significantly affect secretion of Hcp. Total (T) and secreted (S) proteins isolated from wild-type C58 and two ΔaopB strains grown in AB-MES (pH 5.5) for 6 h at 25°C were separated by 12% Glycine-SDS-PAGE and examined by western [file pone.0067647.s001.pdf]

Supporting Information

Systematic dissection of the *Agrobacterium* type VI secretion system reveals machinery and secreted components for subcomplex formation

Jer-Sheng Lin[1], Lay-Sun Ma[1,2,3], and Erh-Min Lai[1,2,3]*

[1] Institute of Plant and Microbial Biology, Academia Sinica, Taipei, Taiwan.

[2] Molecular and Biological Agricultural Sciences Program, Taiwan International Graduate Program, National Chung-Hsing University and Academia Sinica, Taipei, Taiwan.

[3] Graduate Institute of Biotechnology, National Chung-Hsing University, Taichung, Taiwan.

Key words: Type VI secretion system, Hemolysin-coregulated protein, phage tail-like structure, protein-protein interaction, *Agrobacterium tumefaciens*

Correspondence:

Dr. Erh-Min Lai

128, Sec. 2, Academia Road, Nankang

Taipei, Taiwan 11529

Tel: 886-2-27871158

Fax: 886-2-27827954

Email: emlai@gate.sinica.edu.tw

Information S1

Plasmid construction and generation of in-frame deletion mutants

The plasmid pJQ200KS-Δ*tssF* was created by ligating the *Sac*I/*Bam*HI-digested *tssF* PCR product 1 (~500 bp DNA fragment upstream of the *tssF* open reading frame [ORF]) and the *BamH*I/*Xma*I-digested *tssF* PCR product 2 (~500 bp DNA fragment downstream of the *tssF* ORF) into *Sac*I/*Xma*I sites of pJQ200KS and used to generate the deletion mutant Δ*tssF* (EML1090). The rest of pJQ200KS derivatives (Supplementary Table S1) were created by ligating the *Xba*I/*Bam*HI-digested PCR product 1 (~500 bp DNA fragments upstream of each target gene) and the *BamH*I/*Xma*I-digested PCR product 2 (~500 bp DNA fragments downstream of each target gene) into *Xba*I/*Xma*I sites of pJQ200KS and used to generate each of the deletion mutants (Supplementary Tables S1 and S2). For each in-frame deletion mutant confirmed by PCR, at least 2 independent colonies were selected to determine its ability in mediating Hcp secretion.

For complementation, the gene of interest containing its ribosomal-binding sequence (RBS) and ORF was cloned to be driven by a *lac* promoter on the broad host range vector pRL662 [1]. The PCR products of *tssK* and *clpV* genes were digested by *Hind*III/*Xba*I and cloned into the same sites of pRL662 to create the plasmids pTssK and pClpV. The PCR-amplified *fha* and *tssE* genes were digested by *BamH*I/*Xba*I and cloned into the same sites of pRL662 to create the plasmids pFha and pTssE. The remaining genes were amplified with primers described in Supplementary Table S2, and the PCR products were digested by *Xho*I/*Xba*I and cloned into the same sites of pRL662 to create the plasmids pTssG, pTssF, pTssC$_{40}$, pTssC$_{41}$, pTssB, pTssA, pHcp, pAtu4346, pAtu4347, pVgrG-1, and pVgrG-2.

To convert each of Δ*fha* and Δ*tssC*$_{41}$ back to the wild type (revertant), pJQ200KS derivatives harboring the *fha* and *tssC*$_{41}$ genes, including their respective upstream and downstream regions, were used for double crossover. The PCR-amplified products were digested by *Xba*I/*Xma*I and cloned into the same sites of pJQ200KS to create the plasmids pJQ200KS-*fha*, and pJQ200KS-*tssC*$_{41}$. The resulting revertants were designated as EML2137 & 2138 (*fha* R-1 and R-2), and EML2141 & 2142 (*tssC*$_{41}$ R-1 and R-2), respectively.

The expression vector pET22b(+) was used to overexpress proteins driven by the T7 promoter via IPTG induction in *E. coli* BL21 (DE3). Each ORF (without stop codon) of *ppkA*, *tssK*, *fha*, *tssE*, *tssC*$_{41}$, *tssB*, *tssA*, *atu4346*, *atu4347*, *atu4349*, *vgrG-1*, *rpoA*, and *aopB* was PCR-amplified with primers described in Supplementary Table S2 and cloned into the same sites of pET22b(+) with appropriate enzyme sites. The *tssB-tssC*$_{41}$ fragment (without stop codon) was PCR-amplified with primers described in Supplementary Table S2 and cloned into the same sites of pET22b(+) with

appropriate enzyme sites to create the plasmid pET-TssB-$TssC_{41}$-His. The *pppA* and *clpV* ORFs (without stop codon) were PCR-amplified, digested by *Hind*III, and cloned into pET22b(+), which was first digested by *NdeI*, followed by Klenow repair, and finally digested by *Hind*III. To construct the pET-N-TssL-His for expressing the N terminus (residues 1 to 255) of TssL, the plasmid pAD-N-TssL [2] was digested by *Nde*I/*Xho*I and cloned into the same sites of pET22b(+).

To construct the plasmids for expressing proteins without tag, each DNA fragment containing the RBS and ORF (with stop codon) of *$tssC_{41}$*, *hcp*, *atu4347*, *vgrG-1*, and *exoR-Sterp* was PCR-amplified with primers described in Supplementary Table S2 and cloned into pTrc200 with appropriate enzyme sites to create the plasmids pTrc-$TssC_{41}$, pTrc-Hcp, pTrc-Atu4347, pTrc-VgrG-1, and pTrc-ExoR-Strep.

The plasmid pTssB-Strep used in Strep-Tag pull down assay was created by PCR amplifying *tssB* ORF with primers described in Supplementary Table S2 and cloned into the *Xho*I/*Xba*I sites of pRL662.

For the constructs used for yeast two-hybrid, the *$tssC_{41}$* and *tssB* ORFs (without stop codon) were PCR-amplified with primers described in Supplementary Table S2, digested by *Nde*I/*BamH*I, and cloned into the same sites of pGBKT7 or pGADT7 to create the plasmids pGBKT7-$TssC_{41}$, pGBKT7-TssB, pGADT7-$TssC_{41}$, and pGADT7-TssB, respectively.

Biochemical fractionation

Isolation of *A. tumefaciens* cellular fractions was as described [2].

Table S1. Bacterial strains and plasmids

Strain /plasmid	Relevant characteristics	Source/ reference
A.tumefaciens		
C58	Wild type virulent strain containing nopaline-type Ti plasmid pTiC58	Eugene Nester
EML1213	Entire promoter region deletion mutant, C58Δ*pro*	This study
EML1218	Entire *imp* operon deletion mutant, C58Δ*imp*	This study
EML1060	*ppkA*(*atu4330*) in frame deletion mutant, C58Δ*ppkA*	This study
EML1063	*pppA* (*atu4331*) in frame deletion mutant, C58Δ*pppA*	This study
EML1068	*tssM* (*atu4332*) in frame deletion mutant, C58Δ*icmF*	[2]
EML1073	*tssL* (*atu4333*) in-frame deletion mutant, C58Δ*icmH*	[2]
EML1078	*tssK* (*atu4334*) in frame deletion mutant, C58Δ*atu4334*	This study
EML1521	*fha* (*atu4335*) in frame deletion mutant, C58Δ*fha*	This study
EML1086	*tssG* (*atu4336*) in-frame deletion mutant, C58Δ*atu4336*	This study
EML1090	*tssF* (*atu4337*) in frame deletion mutant, C58Δ*atu4337*	This study
EML1093	*tssE* (*atu4338*) in frame deletion mutant, C58Δ*atu4338*	This study
EML1097	*tagJ* (*atu4339*) in frame deletion mutant, C58Δ*atu4339*	This study
EML1100	$tssC_{40}$ (*atu4340*) in frame deletion mutant, C58Δ*atu4340*	This study
EML1105	$tssC_{41}$ (*atu4341*) in frame deletion mutant, C58Δ*vipB*	This study
EML1109	*tssB* (*atu4342*) in frame deletion mutant, C58Δ*vipA*	This study
EML1113	*tssA* (*atu4343*) in-frame deletion mutant, C58Δ*atu4343*	This study
EML1117	*clpV* (*atu4344*) in frame deletion mutant, C58Δ*clpV*	This study

EML1122	*hcp* (*atu4345*) in frame deletion mutant, C58Δ*hcp*	This study
EML1127	*atu4346* in frame deletion mutant, C58Δ*atu4346*	This study
EML1131	*atu4347* in frame deletion mutant, C58Δ*atu4347*	This study
EML1134	*vgrG-1* (*atu4348*) in frame deletion mutant, C58Δ*vgrG-1*	This study
EML1137	*atu4349* in frame deletion mutant, C58Δ*atu4349*	This study
EML1142	*atu4350* in frame deletion mutant, C58Δ*atu4350*	This study
EML1145	*atu4352* in frame deletion mutant, C58Δ*atu4352*	This study
EML1166	*vgrG-2* (*atu3642*) in frame deletion mutant, C58Δ*vgrG-2*	This study
EML1289	*vgrG-1* and *vgrG-2* double in frame deletion mutant, C58Δ*vgrG-1*/*-2*	This study
EML3553	*atu4346* and *atu4347* double in frame deletion mutant, C58Δ*atu4346*Δ*atu4347*	This study
EML3700	*aopB* in frame deletion mutant, C58Δ*aopB*	This study
EML2137	Complementation of *fha* gene to linear chromosome of Δ*fha* strain, revertant strain of Δ*fha*-1	This study
EML2138	Complementation of *fha* gene to linear chromosome of Δ*fha* strain, revertant strain of Δ*fha*-2	This study
EML2141	Complementation of $tssC_{41}$ gene to linear chromosome of $\Delta tssC_{41}$ strain, revertant strain of $\Delta tssC_{41}$-1	This study
EML2142	Complementation of $tssC_{41}$ gene to linear chromosome of $\Delta tssC_{41}$ strain, revertant strain of $\Delta tssC_{41}$-2	This study
EML829	Δ*actCBA*, deletion of *actCBA* in NT1RE	[4]
E. coli		
DH10B	Host for DNA cloning	Invitrogen
BL21(DE3)	Host for overexpressing proteins driven by T7 promoter	[5]
S. cerevisiae		
AH109	Host for yeast two-hybrid analysis	Clontech
**Plasmids**		
pRL662	Gm^r, broad-host range vector derived from pBBR1MCS-2	[1]

pET22b(+)	Ap^r, *E. coli* overexpression vector to generate C-terminal His-tagged protein	Novagen
pJQ200KS	Gm^r, suicide plasmid containing Gm^r and *sacB* gene for selection of double crossover	[6]
pTrc200	Sp^R , pVS1 origin *lacI*^{q}, *trc* promoter expression vector	[7]
pGADT7	Ap^r, AD vector used in yeast two-hybrid assay	Clontech
pGBKT7	Km^r, DNA-BD vector used in yeast-two hybrid assay	Clontech
pTssM	Gm^r, pRL662 expressing TssM driven by *lacZp*	[3]
pTssL	Gm^r, pRL662 expressing TssL driven by *lacZp*	[2]
pTssK	Gm^r, pRL662 expressing TssK driven by *lacZp*	This study
pFha	Gm^r, pRL662 expressing Fha driven by *lacZp*	This study
pTssG	Gm^r, pRL662 expressing TssG driven by *lacZp*	This study
pTssF	Gm^r, pRL662 expressing TssF driven by *lacZp*	This study
pTssE	Gm^r, pRL662 expressing TssE driven by *lacZp*	This study
$pTssC_{40}$	Gm^r, pRL662 expressing $TssC_{40}$ driven by *lacZp*	This study
$pTssC_{41}$	Gm^r, pRL662 expressing $TssC_{41}$ driven by *lacZp*	This study
pTssB	Gm^r, pRL662 expressing TssB driven by *lacZp*	This study
pTssA	Gm^r, pRL662 expressing TssA driven by *lacZp*	This study
pClpV	Gm^r, pRL662 expressing ClpV driven by *lacZp*	This study
pHcp	Gm^r, pRL662 expressing Hcp driven by *lacZp*	This study
pAtu4346	Gm^r, pRL662 expressing Atu4346 driven by *lacZp*	This study
pAtu4347	Gm^r, pRL662 expressing Atu4347 driven by *lacZp*	This study
pVgrG-1	Gm^r, pRL662 expressing VgrG-1 driven by *lacZp*	This study
pVgrG-2	Gm^r, pRL662 expressing VgrG-2 driven by *lacZp*	This study
pTssB-Strep	Gm^r, pRL662 expressing TssB-Strep fusion protein driven by *lacZp*	This study
pET-PpkA-His	Ap^r, pET22b overexpressing His-tagged PpkA in *E coli*	This study
pET-PppA-His	Ap^r, pET22b overexpressing His-tagged PppA in *E coli*	This study

pET-N-TssL-His	Ap^r, pET22b overexpressing His-tagged N terminus of TssL (residues 1 to 255) in *E. coli*	This study
pET-TssK-His	Ap^r, pET22b overexpressing His-tagged TssK in *E coli*	This study
pET-Fha-His	Ap^r, pET22b overexpressing His-tagged Fha in *E coli*	This study
pET-TssE-His	Ap^r, pET22b overexpressing His-tagged TssE in *E coli*	This study
pET-$TssC_{41}$-His	Ap^r, pET22b overexpressing His-tagged $TssC_{41}$ in *E coli*	This study
pET-TssB-His	Ap^r, pET22b overexpressing His-tagged TssB in *E coli*	This study
pET-TssA-His	Ap^r, pET22b overexpressing His-tagged TssA in *E coli*	This study
pET-ClpV-His	Ap^r, pET22b overexpressing His-tagged ClpV in *E coli*	This study
pET-Hcp-His	Ap^r, pET22b overexpressing His-tagged Hcp in *E coli*	[3]
pET-Atu4346-His	Ap^r, pET22b overexpressing His-tagged Atu4346 in *E coli*	This study
pET-Atu4347-His	Ap^r, pET22b overexpressing His-tagged Atu4347 in *E coli*	This study
pET-VgrG-1-His	Ap^r, pET22b overexpressing His-tagged VgrG-1 in *E coli*	This study
pET-TssB-$TssC_{41}$-His	Ap^r, pET22b overexpressing TssB and His-tagged $TssC_{41}$ in *E coli*	This study
pET-RpoA-His	Ap^r, pET22b overexpressing His-tagged Atu1923 (RpoA) in *E coli*	This study
pET-AopB-His	Ap^r, pET22b overexpressing His-tagged Atu1131 (AopB) in *E coli*	This study
pJQ200KS-Δ*pro*	Gm^r, used in generating entire promoter region deletion mutant of *A. tumefaciens* C58	This study
pJQ200KS-Δ*imp*	Gm^r, used in generating entire *imp* operon deletion mutant of *A. tumefaciens* C58	This study
pJQ200KS-Δ*ppkA*	Gm^r, used in generating *ppkA* in-frame deletion mutant of *A. tumefaciens* C58	This study
pJQ200KS-Δ*pppA*	Gm^r, used in generating *pppA* in-frame deletion mutant of *A. tumefaciens* C58	This study

pJQ200KS-Δ*tssK*	Gm^r, used in generating *tssK* in-frame deletion mutant of *A. tumefaciens* C58	This study
pJQ200KS-Δ*fha*	Gm^r, used in generating *fha* in-frame deletion mutant of *A. tumefaciens* C58	This study
pJQ200KS-Δ*tssG*	Gm^r, used in generating *tssG* in-frame deletion mutant of *A. tumefaciens* C58	This study
pJQ200KS-Δ*tssF*	Gm^r, used in generating *tssF* in-frame deletion mutant of *A. tumefaciens* C58	This study
pJQ200KS-Δ*tssE*	Gm^r, used in generating *tssE* in-frame deletion mutant of *A. tumefaciens* C58	This study
pJQ200KS-Δ*tagJ*	Gm^r, used in generating *tagJ* in-frame deletion mutant of *A. tumefaciens* C58	This study
pJQ200KS-Δ$tssC_{40}$	Gm^r, used in generating $tssC_{40}$ in-frame deletion mutant of *A. tumefaciens* C58	This study
pJQ200KS-Δ$tssC_{41}$	Gm^r, used in generating $tssC_{41}$ in-frame deletion mutant of *A. tumefaciens* C58	This study
pJQ200KS-Δ*tssB*	Gm^r, used in generating *tssB* in-frame deletion mutant of *A. tumefaciens* C58	This study
pJQ200KS-Δ*tssA*	Gm^r, used in generating *tssA* in-frame deletion mutant of *A. tumefaciens* C58	This study
pJQ200KS-Δ*clpV*	Gm^r, used in generating *clpV* in-frame deletion mutant of *A. tumefaciens* C58	This study
pJQ200KS-Δ*hcp*	Gm^r, used in generating *hcp* in-frame deletion mutant of *A. tumefaciens* C58	This study
pJQ200KS-Δ*atu4346*	Gm^r, used in generating *atu4346* in-frame deletion mutant of *A. tumefaciens* C58	This study
pJQ200KS-Δ*atu4347*	Gm^r, used in generating *atu4347* in-frame deletion mutant of *A. tumefaciens* C58	This study
pJQ200KS-Δ*vgrG-1*	Gm^r, used in generating *vgrG-1* in-frame deletion mutant of *A. tumefaciens* C58	This study
pJQ200KS-Δ*atu4349*	Gm^r, used in generating *atu4349* in-frame deletion mutant of *A. tumefaciens* C58	This study
pJQ200KS-Δ*atu4350*	Gm^r, used in generating *atu4350* in-frame deletion mutant of *A. tumefaciens* C58	This study
pJQ200KS-Δ*atu4352*	Gm^r, used in generating *atu4352* in-frame deletion mutant of *A. tumefaciens* C58	This study
pJQ200KS-Δ*vgrG-2*	Gm^r, used in generating *vgrG-2* in-frame deletion mutant of *A. tumefaciens* C58	This study

pJQ200KS-Δ*46*Δ*47*	Gm^r, used in generating *atu4346* and *atu4347*double in-frame deletion mutant of *A. tumefaciens* C58	This study
pJQ200KS-Δ*aopB*	Gm^r, used in generating *aopB* in-frame deletion mutant of *A. tumefaciens* C58	This study
pJQ200KS-*fha*	Gm^r, used in generating revertant strain of Δ*fha*	This study
pJQ200KS-$tssC_{41}$	Gm^r, used in generating revertant strain of Δ$tssC_{41}$	This study
pTrc-$TssC_{41}$	Sp^R, pTrc200 expressing $TssC_{41}$ without tag	This study
pTrc-Hcp	Sp^R, pTrc200 expressing Hcp without tag	This study
pTrc-Atu4347	Sp^R, pTrc200 expressing Atu4347 without tag	This study
pTrc-VgrG-1	Sp^R, pTrc200 expressing VgrG-1 without tag	This study
pTrc-ExoR-Strep	Sp^R, pTrc200 expressing ExoR-Strep fusion protein	This study
pGBKT7-$TssC_{41}$	Km^r, DNA-BD vector expressing $TssC_{41}$	This study
pGBKT7-TssB	Km^r, DNA-BD vector expressing TssB	This study
pGBKT7-53	Km^r, DNA-BD vector expressing murine p53	Clontech
pGADT7-$TssC_{41}$	AP^r, AD vector expressing $TssC_{41}$	This study
pGADT7-TssB	AP^r, AD vector expressing TssB	This study
pGADT7-T	Ap^r, AD vector expressing SV40 large T-antigen	Clontech

Table S2. Primers used in this study

Primer	Plasmids	Sequence (5 '-3')[a]	Source / reference
1. Promoter deletion 1F-XbaI	pJQ200KS-Δ*pro*	5'-GC**TCTAGA**GCCTCTCCTGAACTTGTCAGC-3'	This study
2. Promoter deletion 1R-BamHI		5'-CG**GGATCC**ATGTCGCATATCGATCTCAATCG-3'	This study
3. Promoter deletion 2F-BamHI		5'-CG**GGATCC**TTGGATACACAGCATGTTAAAAG-3'	This study
4. Promoter deletion 2R-XmaI		5'-TCCC**CCCGGG**GCTATCCGGTACAGTTCTTCG-3'	This study
5. Imp deletion 1F-XbaI	pJQ200KS-Δ*imp*	5'-GC**TCTAGA**CTGCCGTGAGGATGTTCTGG-3'	This study
6. Imp deletion 1R-BamHI		5'-CG**GGATCC**CAAGAGTAGTCTATCCCCAG-3'	This study
7. Imp deletion 2F-BamHI		5'-CG**GGATCC**CTGTAGCGCCGGCGTCAGTTG-3'	This study
8. Imp deletion 2R-XmaI		5'-TCCC**CCCGGG**CGGGAAGACCGTCAGAACATCC-3 '	This study
9. PpkA 1F-XbaI	pJQ200KS-Δ*ppkA*	5'-GC**TCTAGA**GGAGATGATGGCACAGCAGATC-3'	This study
10. PpkA 1R-BamHI		5'-CG**GGATCC**CCGCATGCCATCATGGCGAATG-3'	This study
11. PpkA 2F-BamHI		5'-CG**GGATCC**CTGTAGCGCCGGCGTCAGTTG-3'	This study
12. PpkA 2R-XmaI		5'-TCCC**CCCGGG**CCCGTCAGGAGCGTGTACTTG-3'	This study
13. PppA 1F-XbaI	pJQ200KS-Δ*pppA*	5'-GC**TCTAGA**GCCCAGTTCGAAAATGCCGAC-3'	This study
14. PppA 1R-BamHI		5'-CG**GGATCC**ATCGGCCATCAGTTGCGATTG-3'	This study
15. PppA 2F-BamHI		5'-CG**GGATCC**GGCTAGACATCCACTTGATGAG-3'	This study
16. PppA 2R-XmaI		5'-TCCC**CCCGGG**CGAAGGATCGAGATCACCTGC-3'	This study
17. TssK 1F-XbaI	pJQ200KS-Δ*tssK*	5'-GC**TCTAGA**GATACAGACGAAGACCCGATC-3'	This study
18. TssK 1R-BamHI		5'-CG**GGATCC**ATGTCTCATCGTGGTTGTTTAC-3'	This study
19. TssK 2F-BamHI		5'-CG**GGATCC**GAATGAGCACGGACAACCCCT-3'	This study
20. TssK 2R-XmaI		5'-TCCC**CCCGGG**TTGTGGAAGGTGGAAACGAGG-3'	This study
21. Fha 1F-XbaI	pJQ200KS-Δ*fha*	5'-GC**TCTAGA**TGCCGAAGACACACTTCTGC-3'	This study
22. Fha 1R-BamHI		5'-CG**GGATCC**CTTCATGTCCTGGCCCTCAC-3'	This study
23. Fha 2F-BamHI		5'-CG**GGATCC**ACATGAGAACCGTGTTGCCTG-3'	This study
24. Fha 2R-XmaI		5'-TCCC**CCCGGG**GGAGGAATGAAATCCGGATCG-3'	This study
25. TssG 1F-XbaI	pJQ200KS-Δ*tssG*	5'-GC**TCTAGA**TGGTAGCGAGCATCGACACG-3'	This study
26. TssG 1R-BamHI		5'-CG**GGATCC**GTCCATCCCGGTCTCCTAAAG-3'	This study
27. TssG 2F-BamHI		5'-CG**GGATCC**GTGTGAGGGCCAGGACATGAA-3'	This study
28. TssG 2R-XmaI		5'-TCCC**CCCGGG**ACGTTGCGCGAAATGGACTTC-3'	This study
29. TssF 1F-SacI	pJQ200KS-Δ*tssF*	5'-G**GAGCTC**GCTCGTCAGTCTGAAGGATC-3'	This study
30. TssF 1R-BamHI		5'-CG**GGATCC**GCCGTCAGCCATTCGTCG-3'	This study
31. TssF 2F-BamHI		5'-CG**GGATCC**CCGTTGCTTTAGGAGACC-3'	This study
32. TssF 2R-XmaI		5'-TCCC**CCCGGG**TCGCACAGCATGGAGGTG-3'	This study
33. TssE 1F-XbaI	pJQ200KS-Δ*tssE*	5'-GC**TCTAGA**TGGCTGCACGCAATGCGTGG-3'	This study
34. TssE 1R-BamHI		5'-CG**GGATCC**ATCAACCACGGGCGATCTGC-3'	This study
35. TssE 2F-BamHI		5'-CG**GGATCC**AATGGCTGACGGCTTCCTCG-3'	This study
36. TssE 2R-XmaI		5'-TCCC**CCCGGG**AACGGCTGGTCAGCTGGACG-3'	This study
37. TagJ 1F-XbaI	pJQ200KS-Δ*tagJ*	5'-GC**TCTAGA**TCTCGGCATCGAGAGCAACG-3'	This study
38. TagJ 1R-BamHI		5'-CG**GGATCC**TGCAGCCATGGCGCGCAGC-3'	This study
39. TagJ 2F-BamHI		5'-CG**GGATCC**CGTGGTTGATCCGCTGGAGC-3'	This study
40. TagJ 2R-XmaI		5'-TCCC**CCCGGG**TCAGCCATTCGTCGCCTCCA-3'	This study
41. TssC$_{40}$ 1F-XbaI	pJQ200KS-Δ*tssC$_{40}$*	5'-GC**TCTAGA**TCAAGGCGATCTACGAAGACG-3'	This study
42. TssC$_{40}$ 1R-BamHI		5'-CG**GGATCC**GATCACCCCGGTCCGTGCTC-3'	This study
43. TssC$_{40}$ 2F-BamHI		5'-CG**GGATCC**GCATGACGCTGCGCGACGA-3'	This study
44. TssC$_{40}$ 2R-XmaI		5'-TCCC**CCCGGG**TCAACCACGGGCGATCTGC-3'	This study
45. TssC$_{41}$ 1F-XbaI	pJQ200KS-Δ*tssC$_{41}$*	5'-GC**TCTAGA**TGCCAAGGGGCATCAAGGAG-3'	This study
46. TssC$_{41}$ 1R-BamHI		5'-CG**GGATCC**AGCGCTCATGTTTCAGTCCTTC-3'	This study
47. TssC$_{41}$ 2F-BamHI		5'-CG**GGATCC**AAGAACTGAGCACGGACCG-3'	This study
48. TssC$_{41}$ 2R-XmaI		5'-TCCC**CCCGGG**AAGTCATGAACCCGGCTGAG-3'	This study
49. TssB 1F-XbaI	pJQ200KS-Δ*tssB*	5'-GC**TCTAGA**CAGTCTCTGGGATTTCCAGC-3'	This study
50. TssB 1R-BamHI		5'-CG**GGATCC**CCTTGGCATAGATGACTGTTG-3'	This study
51. TssB 2F-BamHI		5'-CG**GGATCC**AGCGAATAATCGCCTGTGCG-3'	This study
52. TssB 2R-XmaI		5'-TCCC**CCCGGG**TCGAAGGAATTGCCGAATTCG-3'	This study
53. TssA 1F-XbaI	pJQ200KS-Δ*tssA*	5'-GC**TCTAGA**GAGCTGCGAAGACGCTCAAG-3'	This study
54. TssA 1R-BamHI		5'-CG**GGATCC**TGTATCCAAGAGTAGTCTATCC-3'	This study
55. TssA 2F-BamHI		5'-CG**GGATCC**AAATAAGCAACTCGGGACGAG-3'	This study
56. TssA 2R-XmaI		5'-TCCC**CCCGGG**TTATTCGCTCGCGCCGTTCT-3'	This study
57. ClpV 1F-XbaI	pJQ200KS-Δ*clpV*	5'-GC**TCTAGA**TCTTCGTCATTATCGTCGCTG-3'	This study
58. ClpV 1R-BamHI		5'-CG**GGATCC**ATGCGACATGCAATGAGCCTC-3'	This study
59. ClpV 2F-BamHI		5'-CG**GGATCC**CATTAAACGAAGGGTAACTCG-3'	This study
60. ClpV 2R-XmaI		5'-TCCC**CCCGGG**TAGGAAGCGATCATCGGAGAC-3'	This study
61. Hcp 1F-XbaI	pJQ200KS-Δ*hcp*	5'-GC**TCTAGA**GGAGCTTACCAAACAGTTCAAG-3'	This study
62. Hcp 1R-BamHI		5'-CG**GGATCC**TGGCATGCTCTAAGCTCCTG-3'	This study
63. Hcp 2F-BamHI		5'-CG**GGATCC**GCTGCTTGATAGCCATGCGAC-3'	This study
64. Hcp 2R-XmaI		5'-TCCC**CCCGGG**TCTGCTCACAGGGAAAGGAG-3'	This study

65. Atu4346 1F-XbaI	pJQ200KS-Δ*atu4346*	5'-GC**TCTAGA**GGTCATTTCAGCTACAGGAGC-3'	This study
66. Atu4346 1R-BamHI		5'-CG**GGATCC**CTGCAATGAAAACGTTCCGGTG-3'	This study
67. Atu4346 2F-BamHI		5'-CG**GGATCC**AACTAGTGGGACGAGCCATG-3'	This study
68. Atu4346 2R-XmaI		5'-TCCC**CCCGGG**GCGAAGACACTTTCAGAACAC-3'	This study
69. Atu4347 1F-XbaI	pJQ200KS-Δ*atu4347*	5'-GC**TCTAGA**CAAGGCTGCTTGATAGCCATG-3'	This study
70. Atu4347 1R-BamHI		5'-CG**GGATCC**GCGCATGGCTCGTCCCACTAG-3'	This study
71. Atu4347 2F-BamHI		5'-CG**GGATCC**TCCTGATTTAGGAGTGTCCCA-3'	This study
72. Atu4347 2R-XmaI		5'-TCCC**CCCGGG**TCCGTCTCGTTAAACTGAACG-3'	This study
73. VgrG-1 1F-XbaI	pJQ200KS-Δ*vgrG-1*	5'-GC**TCTAGA**ATGCGCGTTAACTTTGACACC-3'	This study
74. VgrG-1 1R-BamHI		5'-CG**GGATCC**GTTCATGGGACACTCCTAAATC-3'	This study
75. VgrG-1 2F-BamHI		5'-CG**GGATCC**ATTTGATTATGAACGACACACC-3'	This study
76. VgrG-1 2R-XmaI		5'-TCCC**CCCGGG**TAAAACGACGCAATCCGCAGG-3'	This study
77. Atu4349 1F-XbaI	pJQ200KS-Δ*atu4349*	5'-GC**TCTAGA**GCATCATGAACACGATCATCG-3'	This study
78. Atu4349 1R-BamHI		5'-CG**GGATCC**GTTCATAATCAAATCCTGACAAAC-3'	This study
79. Atu4349 2F-BamHI		5'-CG**GGATCC**GCATGAGTGCGACGACAACTG-3'	This study
80. Atu4349 2R-XmaI		5'-TCCC**CCCGGG**TTCGCAGCCTGTTCCTTATAG-3'	This study
81. Atu4350 1F-XbaI	pJQ200KS-Δ*atu4350*	5'-GC**TCTAGA**CAATCCTGACAAGGCCACAGC-3'	This study
82. Atu4350 1R-BamHI		5'-CG**GGATCC**ACTCATGCGGGCGCTCCGGA-3'	This study
83. Atu4350 2F-BamHI		5'-CG**GGATCC**TCTTGAGCAGTTTAATATTCTTG-3'	This study
84. Atu4350 2R-XmaI		5'-TCCC**CCCGGG**ATAAGCTTACCTTTCTCGTCC-3'	This study
85. Atu4352 1F-XbaI	pJQ200KS-Δ*atu4352*	5'-GC**TCTAGA**TGGAAGCAAGGCTATTTTCAG-3'	This study
86. Atu4352 1R-BamHI		5'-CG**GGATCC**GACCATTTAAATATCCCTCTAG-3'	This study
87. Atu4352 2F-BamHI		5'-CG**GGATCC**GAGTGACGACGATATCCAGC-3'	This study
88. Atu4352 2R-XmaI		5'-TCCC**CCCGGG**CTCGATCTTGAAATCACCGAG-3'	This study
89. VgrG-2 1F-XbaI	pJQ200KS-Δ*vgrG-2*	5'-GC**TCTAGA**TCGCTGAGTGATCGCCATCG-3'	This study
90. VgrG-2 1R-BamHI		5'-CG**GGATCC**ATTCATCAGGAACCTCGATAGC-3'	This study
91. VgrG-2 2F-BamHI		5'-CG**GGATCC**AACTGAAGGAGGCCGGGTCTT-3'	This study
92. VgrG-2 2R-XmaI		5'-TCCC**CCCGGG**CAGAAGATTGCGACGCTCCAC-3'	This study
93. AopB 1F-XbaI	pJQ200KS-Δ*aopB*	5'-GC**TCTAGA**TGTGTCAGATCTGCCTGAACG-3'	This study
94. AopB 1R-BamHI		5'-CG**GGATCC**ACGCATGTTATTCTCCTTTCAG-3'	This study
95. AopB 2F-BamHI		5'-CG**GGATCC**TTCTGATCCTTCTCGGATCG-3'	This study
96. AopB 2R-XmaI		5'-TCCC**CCCGGG**TGACAGCGTGACGATCATGC-3'	This study
97. TssK comN-HindIII	pTssK	5'-CCC**AAGCTT**GTCCGCACGGGAGACTGACG-3'	This study
98. TssK comC-XbaI		5'-GC**TCTAGA**CGGCAAATCCTGCCAGGAAG-3'	This study
99. Fha comN-BamHI	pFha	5'-CG**GGATCC**CGATGCTGTTGTGACCGAGC-3'	This study
100. Fha comC-XbaI		5'-GC**TCTAGA**ATGCCCTCGCTCCAGGCAAC-3'	This study
101. TssG comN-XhoI	pTssG	5'-CCG**CTCGAG**TGGCCATTCCAAACCAATTGC-3'	This study
102. TssG comC-XbaI		5'-GC**TCTAGA**TTGAGTGCAAGCTTCATGTCC-3'	This study
103. TssF comN-XhoI	pTssF	5'-CCG**CTCGAG**CAGCTTTGAATCCCGCATCG-3'	This study
104 .TssF comC-XbaI		5'-GC**TCTAGA**TGGTTGGCATTTGCTGGTCC-3'	This study
105. TssE comN-BamHI	pTssE	5'-CG**GGATCC**ATGTGGTGCCGTTTCACGAG-3'	This study
106. TssE comC-XbaI		5'-GC**TCTAGA**GCTCGTCATTGTATTTTTCGAG-3'	This study
107. TssC$_{40}$ comN-XhoI	pTssC$_{40}$	5'-CCG**CTCGAG**AGAACTGACGACCTCGCTGC-3'	This study
108. TssC$_{40}$ comC-XbaI		5'-GC**TCTAGA**AGCTGGTTATCGTCCAGGAG-3'	This study
109. TssC$_{41}$ comN-XhoI	pTssC$_{41}$ (109&111),	5'-CCG**CTCGAG**GGCATTTTCAAAGCCGCATG-3'	This study
110. TssC$_{41}$ comN-NcoI	pTrc-TssC$_{41}$	5'-CATG**CCATGG**GGCATTTTCAAAGCCGCATG-3'	This study
111. TssC$_{41}$ comC-XbaI	(110&111)	5'-GC**TCTAGA**CTGATCACCCCGGTCCGTG-3'	This study
112. TssB comN-XhoI	pTssB (112&113),	5'-CCG**CTCGAG**GAGACAATAACGGGAAATAAGC-3'	This study
113. TssB comC-XbaI	pTssB-Strep	5'-GC**TCTAGA**GGAGGTTTTCCTCCGCACAG-3'	This study
114. TssB Strep comC-XbaI	(112&114)	5'-GC**TCTAGA**TTACTTTTCGAACTGCGGGTGGCTCC ATTCGCTCGCGCCGTTCTTTTCC-3'	This study
115. TssA comN-XhoI	pTssA	5'-CCG**CTCGAG**CCTCACGTAAGGGAAGTAAC-3'	This study
116. TssA comC-XbaI		5'-GC**TCTAGA**CTTGGCATAGATGACTGTTGC-3'	This study
117. ClpV comN-HindIII	pClpV	5'-CCC**AAGCTT**TATTCCACGTCACGTTGTGG-3'	This study
118. ClpV comC-XbaI		5'-GC**TCTAGA**CCTGTAGCTGAAATGACCTC-3'	This study
119. Hcp comN-XhoI	pHcp (119&121),	5'-CCG**CTCGAG**TAACTCGGCCTCGGAGCAAG-3'	This study
120. Hcp F1	pTrc-Hcp	5'-CATG**CCATGG** TAACTCGGCCTCGGAGCAAG-3'	This study
121. Hcp comC-XbaI	(120&121)	5'-GC**TCTAGA**CGACGTGAGCTGCAAACTGC-3'	This study
122. Atu4346 comN-XhoI	pAtu4346	5'-CCG**CTCGAG**TGCTTGATAGCCATGCGACC-3'	This study
123. Atu4346 comC-XbaI		5'-GC**TCTAGA**GTCAAAGTTAACGCGCATGG-3'	This study
124. Atu4347 comN-XhoI	pAtu4347 (124&126),	5'-CCG**CTCGAG**AACAGGCAGCAACCCTCGTG-3'	This study
125. Atu4347 BamHI F	pTrc-Atu4347	5'-CGC**GGATCC**ACTAGTGGGACGAGCCATGC-3'	This study
126. Atu4347 comC-XbaI	(125&126)	5'-GC**TCTAGA**CAGAAACCGAAGGCTGGTCG-3'	This study
127. VgrG-1 comN-XhoI	pVgrG-1 (127&129),	5'-CCG**CTCGAG**CGGAAACGATCTGGTTCTGG-3'	This study
128. VgrG-1 comN-NcoI	pTrc-VgrG-1 (128&129)	5'-CATG**CCATGG**CGGAAACGATCTGGTTCTGG-3'	This study
129. VgrG-1 comC-XbaI		5'-GC**TCTAGA**TCAATTATGGGTGTGTCGTTC-3'	This study

130. VgrG-2 comN-XhoI	pVgrG-2	5'-CCG**CTCGAG**CGAGATTTGACAGCTGATTG-3'	This study
131. VgrG-2 comC-XbaI		5'-GC**TCTAGA**GGTTCTGAAGTTCCAAGACC-3'	This study
132. PpkA F-NdeI	pET-PpkA-His	5'-GAA**CATATG**CGGGAAAGAAGCGATCAG-3'	This study
133. PpkA R-XhoI		5'-CCG**CTCGAG**CAGAAGCTGTAGCGCTTCCG-3'	This study
134. PppA F	pET-PppA-His	5'-TGGCCGATCAGGCATCAAGG-3'	This study
135. PppA R-HindIII		5'-CCC**AAGCTT**GCCTTGCTCACGCCGGTTTC-3'	This study
136. TssK F-NdeI	pET-TssK-His	5'-GAA**CATATG**AGACATGAGAACCGTGTTG-3'	This study
137. TssK R-HindIII		5'-CCC**AAGCTT**TTCGCGTAACGCCCACATTTC-3'	This study
138. Fha F-NdeI	pET-Fha-His	5'-GAA**CATATG**AAGCTTGCACTCAAGAACAC-3'	This study
139. Fha R-SalI		5'-CGC**GTCGAC**TGTCTCATCGTGGTTGTTTACC-3'	This study
140. TssE F-NdeI	pET-TssE-His	5'-GAA**CATATG**GTTGATCCGCTGGAGCAATATC-3'	This study
141. TssE R-SalI		5'-CGC**GTCGAC**GCCATTCGTCGCCTCCACCAAG-3'	This study
142. $TssC_{41}$ F-NdeI	pET-$TssC_{41}$-His	5'-GAA**CATATG**AGCGCTGAAAGCCTGC-3'	This study
143. $TssC_{41}$ R-XhoI-a	pET-TssB-$TssC_{41}$-His (144&143)	5'-CCG**CTCGAG**GTTCTTGGACGGAATACG-3'	This study
144. TssB F-NdeI	pET-TssB-His	5'-GAA**CATATG**CCAAGGGGCATCAAGGA-3'	This study
145. TssB R-XhoI	pET-TssB-$TssC_{41}$-His (144&143)	5'-CCG**CTCGAG**TTCGCTCGCGCCGTTCTTTTC-3'	This study
146. TssA F-NdeI	pET-TssA-His	5'-GAA**CATATG**GATACACAGCATGTTAAAAGAG-3'	This study
147. TssA R-XhoI		5'-CCG**CTCGAG**TTTCCCGTTATTGTCTCCGC-3'	This study
148. ClpV F	pET-ClpV-His	5'-TGTCGCATATCGATCTCAATCG-3'	This study
149. ClpV R-HindIII		5'-CCC**AAGCTT**ATGCCGCATGCGTCGGGTAGC-3'	This study
150. Atu4346 F-NdeI	pET-Atu4346-His	5'-GAA**CATATG**CAGTTTGCAGCTCACGTCGTG-3'	This study
151. Atu4346 R-XhoI		5'-CCG**CTCGAG**GTTACTTTTCTGCTCACAGGGA-3'	This study
152. Atu4347 F-NdeI	pET-Atu4347-His	5'-GAA**CATATG**CGCGTTAACTTTGACACC-3'	This study
153. Atu4347 R-XhoI		5'-CCG**CTCGAG**GGACCCGCGGCTGGCCTGCA-3'	This study
154. Atu4349 F-NdeI	pET-Atu4349-His	5'-GAA**CATATG**AACGACACACCCATAATTG-3'	This study
155. Atu4349 R-XhoI		5'-CCG**CTCGAG**TGCGGGCGCTCCGGATGCTG-3'	This study
156. VgrG-1 F-NdeI	pET-VgrG-1-His	5'-GAA**CATATG**AACGACCAGCCTTCGGTTTC-3'	This study
157. VgrG-1 R-XhoI		5'-CCG**CTCGAG**AATCCTGACAAACGGTGTGGAA-3'	This study
158. RpoA F-NdeI	pET-RpoA-His	5'-GAA**CATATG**ATTCAGAAGAACTGGCAGG-3'	This study
159. RpoA R-XhoI		5'-CCG**CTCGAG**GTATTGGTCTTCGTAACGCTTTG-3'	This study
160. AopB F-NdeI	pET-AopB-His	5'-GAA**CATATG**ATGCGTATTTTCGTAGCAACC -3'	This study
161. AopB R-XhoI		5'-CCG**CTCGAG**GAACTTGACGCCGATACCG-3'	This study
162. ExoR F (XmaI)	pTrC-ExoR-Strep	5'-G**CCCGGG**AGTGAGAGAAGTTCGCCGAAATGC-3'	[8]
163. ExoR C-Strep R-XbaI		5'-GC**TCTAGA**TCACTTTTCGAACTGCGGGTGGCTCC AATCCGGATCGTTGAACTGCATG-3'	This study
164. $TssC_{41}$ R-BamHI	pGBKT7-$TssC_{41}$ (142&164) pGADT7-$TssC_{41}$ (142&164)	5'-CG**GGATCC**GTTCTTGGACGGAATACG-3'	This study
165. TssB-BamHI	pGBKT7-TssB (144&165) pGADT7-TssB (144&165)	5'-CG**GGATCC**TTCGCTCGCGCCGTTCTTTTC-3'	This study
166. Atu4329 RT1		5'-GGAAGACCGTCAGAACATCC-3'	This study
167. Atu4329 RT2-BamHI		5'-CG**GGATCC**GACGTGATAGATCACCGGTC-3'	This study
168. PpkA-RT1		5'-CAGGTGATCTCGATCCTTCG-3'	This study
169. PpkA-RT2-BamHI		5'-CG**GGATCC**GTCCCACGGCGTATATGTCC-3'	This study
170. TssG-RT1		5'-GGGAACAGGACAACGGTTTC-3'	This study
171. TssG-RT2-BamHI		5'-CG**GGATCC**TGTCCGAGCTGGCTTTGTTC-3'	This study
172. TssA-RT1		5'-CATGTCTTCCCATCTGGCAG-3'	This study
173. TssA-RT2-BamHI		5'-CG**GGATCC**GAGTTTCAAACGCCTCATCG-3'	This study
174. ClpV-RT1		5'-AGAAACTGCGCAATGTCTGC-3'	This study
175. ClpV-RT2-BamHI		5'-CG**GGATCC**TGCGGGCTTAAGAATATTCG-3'	This study
176. VgrG-1-RT1		5'-ACCGTAACCACATCAACGACTG-3'	This study
177. VgrG-1-RT2-BamHI		5'-CG**GGATCC**AATACGCGTGGTCGATGAGC-3'	This study
178. Atu4352-RT1		5'-CACGCTGAAAGGTCATATGC-3'	This study
179. Atu4352-RT2-BamHI		5'-CG**GGATCC**GTATGGTAGCGACCGATGAG-3'	This study
180. Atu4353 RT1		5'-TCCGCATCCATGGGAATAGC-3'	This study
181. Atu4353 RT2-BamHI		5'-CG**GGATCC**TTCCGTCGCGTATATCCACG-3'	This study
182. 16S-F		5'-ACGCTGGCGGCAGGCTTAACACAT-3'	[9]
183. 16S-R		5'-TAAGCCGCCTTCGCCACTGGTGTT-3'	[9]
184.46/47 1F-XbaI	pJQ200KS-Δ*46*Δ*47*	5'-GC**TCTAGA**GGTCATTTCAGCTACAGGAGC-3'	This study
185.46/47 1R-BamHI		5'-AACG**GGATCC**GCGCATGAAAAACCCGCCTC-3'	This study
186.46/47 2F-BamHI		5'-CG**GGATCC**TCCTGATTTAGGAGTGTCCCA-3'	This study
187.46/47 2R-XmaI		5'-TCCC**CCCGGG**TCCGTCTCGTTAAACTGAACG-3'	This study

a: Restriction enzyme sites are underlined in bold face.

Table S3. Characteristics of proteins encoded by the *imp* cluster.

Gene name<br>Protein name	Conserved<br>Ortholog[a]	Molecular<br>weight[b]<br>(Da) / pI	Essential for<br>Hcp<br>secretion[c]	Predicted cellular<br>localization[d, e]	Predicted<br>signal<br>peptide[f, g]	Predicted<br>non-classically<br>secreted protein[h]
atu4330<br>PpkA<br>TagE	V	270 a.a.<br>29,472 / 6.71	−/+	1.[d] Unknown<br>2.[e] C (cytoplasmic)	1.[f] No SP<br>2.[g] No SP	No<br>(0.08)
atu4331<br>PppA<br>TagF	V	471 a.a.<br>51,714 / 6.39	−	1. Cytoplasmic<br>membrane<br>2. C (cytoplasmic)	1. No SP<br>2. No SP	No<br>(0.14)
atu4332<br>TssM	V	1159 a.a.<br>128,315 /<br>6.27	+	1. Cytoplasmic<br>membrane<br>2. IM (inner<br>membrane ptotein<br>with 3 TM)(26-48,<br>57-79, 438-460)[g]	1. No SP<br>2. No SP	No<br>(0.12)
atu4333<br>TssL	V	501 a.a.<br>55,188 / 5.85	+	1. Cytoplasmic<br>membrane<br>2. IM (inner<br>membrane protein<br>with 1<br>TM)(254-276) [g]	1. No SP<br>2. No SP	No<br>(0.19)
atu4334<br>TssK	V	446 a. a.<br>49,659 / 5.51	+	1. Cytoplasmic<br>2. C (cytoplasmic)	1. No SP<br>2. No SP	No<br>(0.10)
atu4335<br>Fha<br>TagH	V	399 a.a.<br>43,399 / 4.85	+	1. Cytoplasmic<br>2. C (cytoplasmic)	1. No SP<br>2. No SP	No<br>(0.19)
atu4336<br>TssG	V	334 a.a<br>36,123 / 9.35	+	1. Unknown<br>2. C (cytoplasmic)	1. No SP<br>2. No SP	No<br>(0.20)
atu4337<br>TssF	V	593 a.a.<br>65,507 / 6.26	+	1. Unknown<br>2. C (cytoplasmic)	1. No SP<br>2. No SP	No<br>(0.11)
atu4338<br>TssE	V	169 a.a.<br>19,002 / 5.93	+	1. Cytoplasmic<br>2. C (cytoplasmic)	1. No SP<br>2. No SP	No<br>(0.29)
atu4339<br>TagJ	V	274 a.a.<br>29,813 / 4.88	−	1. Cytoplasmic<br>2. C (cytoplasmic)	1. No SP<br>2. No SP	No<br>(0.09)
atu4340<br>TssC$_{40}$	V	464 a.a.<br>50,949 / 5.51	+	1. Cytoplasmic<br>2. C (cytoplasmic)	1. No SP<br>2. No SP	No<br>(0.16)

atu4341<br>$TssC_{41}$	V	493 a.a.<br>55,032 / 5.44	+	1. Cytoplasmic<br>2. C (cytoplasmic)	1. No SP<br>2. No SP	No<br>(0.39)
atu4342<br>TssB	V	169 a.a.<br>19,098 / 5	+	1. Cytoplasmic<br>2. C (cytoplasmic)	1. No SP<br>2. No SP	No<br>(0.10)
atu4343<br>TssA	V	351 a.a.<br>38,491 / 5.14	+	1. Unknown<br>2. C (cytoplasmic)	1. No SP<br>2. No SP	No<br>(0.08)
atu4344<br>ClpV<br>TssH	V	892 a.a.<br>96,506 / 5.09	+	1. Cytoplasmic<br>2. C (cytoplasmic)	1. No SP<br>2. No SP	No<br>(0.09)
atu4345<br>Hcp<br>TssD	V	158 a.a.<br>17,316 / 5.12	+	1. Unknown<br>2. EC (extracellular)	1. No SP<br>2. No SP	Yes<br>(Score 0.947)
atu4346<br>Atu4346		129 a.a.<br>13,737 / 4.93	−	1. Unknown<br>2. P (periplasm)	1. Yes(1-25)<br>2. Yes(1-25)	Yes<br>(Score 0.539)
atu4347<br>Atu4347		166 a.a.<br>18,271 / 9.05	−	1. Unknown<br>2. OM (outer membrane)	1. No SP<br>2. No SP	Yes<br>(Score 0.941)
atu4348<br>VgrG-1<br>TssI-1	V	816 a.a.<br>88,523 / 5.98	(+)	1. Cytoplasmic<br>2. C (cytoplasmic)	1. No SP<br>2. No SP	Yes<br>(Score 0.624)
atu4349<br>Atu4349		318 a.a<br>34,604 / 5.24	−	1. Unknown<br>2. C (cytoplasmic)	1. No SP<br>2. No SP	Yes<br>(Score 0.733)
atu4350<br>Atu4350		278 a.a.<br>29,511 / 5.17	−	1. Periplasmic<br>2. OM (outer membrane)	1. No SP<br>2. Yes(1-37)	Yes<br>(Score 0.923)
atu4351<br>Atu4351		224 a.a.<br>24,945 / 7.74	NA	1. Unknown<br>2. C (cytoplasmic)	1. No SP<br>2. No SP	No<br>(0.11)
atu4352<br>Atu4352	V	101 a.a.<br>10,371 / 7.79	−	1. Unknown<br>2. EC (extracellular)	1. No SP<br>2. No SP	Yes<br>(Score 0.878)
atu3642<br>VgrG-2<br>TssI-2	V	754 a.a.<br>81,764 /5.8	(+)	1. Cytoplasmic<br>2. C (cytoplasmic)	1. No SP<br>2. No SP	Yes<br>(Score 0.598)

a.a.: amino acid.

a: Conserved orthologs: These data have been reported elsewhere [10-12].

b: The molecular weight and isoelectric point (pI) are based on prediction by the software ExPASy (http://www.expasy.ch/tools/pi_tool.html).

c: Essential for Hcp secretion is based on experimental data presented in Fig. 2

d: The cellular localization is based on prediction by the software PSORTb (http://www.psort.org/psortb/index.html).

e: The cellular localization is based on prediction by the software SOSUIGramN (http://bp.nuap.nagoya-u.ac.jp/sosui/sosuigramn/sosuigramn_submit.html).

f: The prediction of signal peptides was by use of the software SignalP (http://www.cbs.dtu.dk/services/SignalP/).

g: The prediction of signal peptides was by use of the software SOSUIsignal (http://bp.nuap.nagoya-u.ac.jp/sosui/sosuisignal/sosuisignal_submit.html).

h: The prediction was by use of the software SecretomeP (http://www.cbs.dtu.dk/services/SecretomeP/). Non-classically secreted proteins should obtain an NN-score exceeding the threshold of 0.5.

References

1. Vergunst AC, Schrammeijer B, den Dulk-Ras A, de Vlaam CM, Regensburg-Tuink TJ, et al. (2000) VirB/D4-dependent protein translocation from *Agrobacterium* into plant cells. Science 290: 979-982.
2. Ma LS, Lin JS, Lai EM (2009) An IcmF family protein, ImpLM, is an integral inner membrane protein interacting with ImpKL, and its walker a motif is required for type VI secretion system-mediated Hcp secretion in *Agrobacterium tumefaciens*. J Bacteriol 191: 4316-4329.
3. Wu HY, Chung PC, Shih HW, Wen SR, Lai EM (2008) Secretome analysis uncovers an Hcp-family protein secreted via a type VI secretion system in *Agrobacterium tumefaciens*. J Bacteriol 190: 2841-2850.
4. Liu AC, Shih HW, Hsu T, Lai EM (2008) A citrate-inducible gene, encoding a putative tricarboxylate transporter, is downregulated by the organic solvent DMSO in *Agrobacterium tumefaciens*. J Appl Microbiol 105: 1372-1383.
5. Studier FW, Rosenberg AH, Dunn JJ, Dubendorff JW (1990) Use of T7 RNA polymerase to direct expression of cloned genes. Methods Enzymol 185: 60-89.
6. Quandt J, Hynes MF (1993) Versatile suicide vectors which allow direct selection for gene replacement in gram-negative bacteria. Gene 127: 15-21.
7. Schmidt-Eisenlohr H, Domke N, Baron C (1999) TraC of IncN plasmid pKM101 associates with membranes and extracellular high-molecular-weight structures in *Escherichia coli*. J Bacteriol 181: 5563-5571.
8. Wu CF, Lin JS, Shaw GC, Lai EM (2012) Acid-induced type VI secretion system is regulated by ExoR-ChvG/ChvI signaling cascade in *Agrobacterium*

tumefaciens. PLoS Pathog 8: e1002938.

9. Lai EM, Shih HW, Wen SR, Cheng MW, Hwang HH, et al. (2006) Proteomic analysis of *Agrobacterium tumefaciens* response to the *vir* gene inducer acetosyringone. Proteomics 6: 4130-4136.
10. Cascales E (2008) The type VI secretion toolkit. EMBO Rep 9: 735-741.
11. Zheng J, Leung KY (2007) Dissection of a type VI secretion system in *Edwardsiella tarda*. Mol Microbiol 66: 1192-1206.
12. Zheng J, Ho B, Mekalanos JJ (2011) Genetic analysis of anti-amoebae and anti-bacterial activities of the type VI secretion system in *Vibrio cholerae*. PLoS One 6: e23876.

Fig. S1

(A)

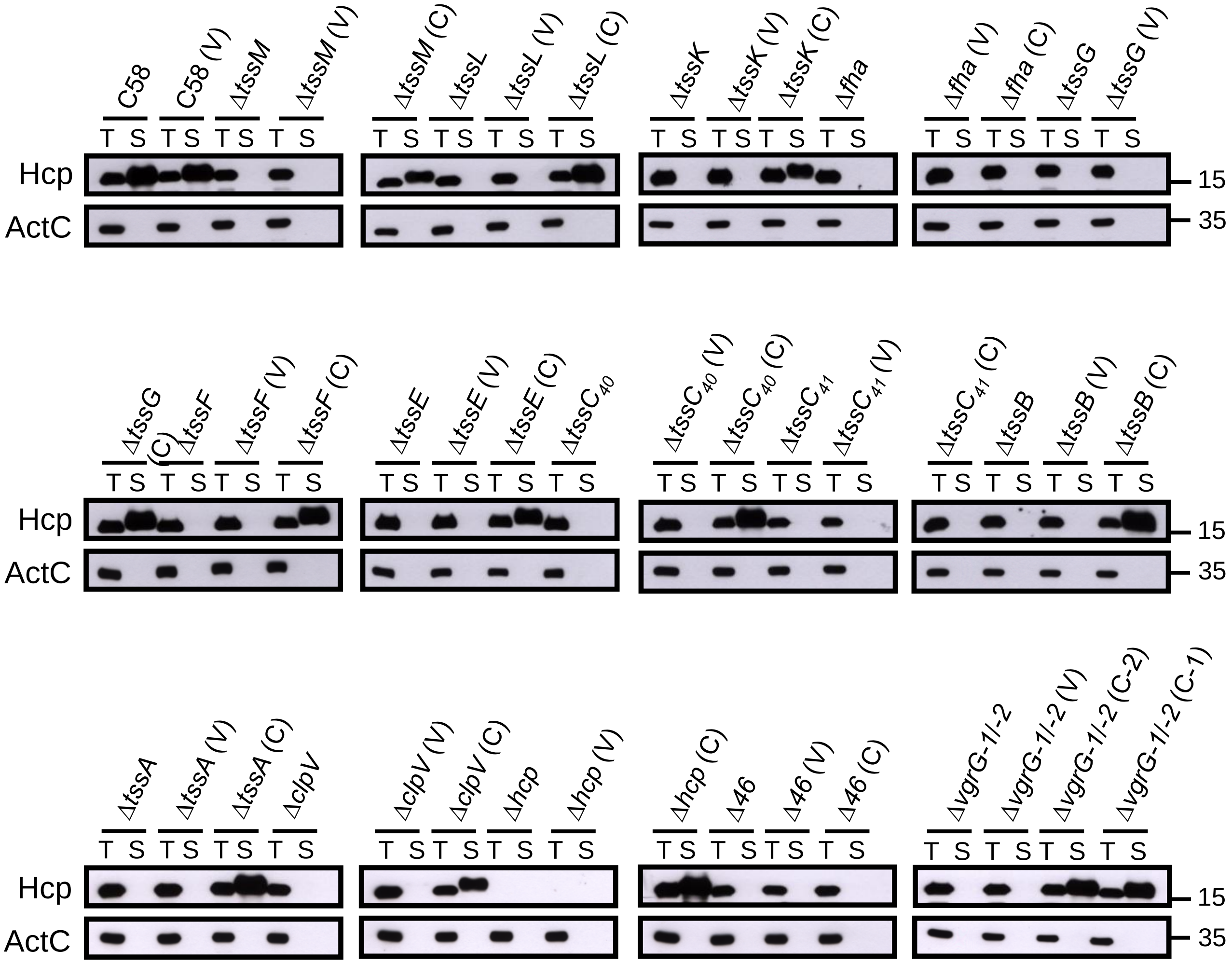
C58
C58 (V)
ΔtssM
ΔtssM (V)
ΔtssM (C)
ΔtssL
ΔtssL (V)
ΔtssL (C)
ΔtssK
ΔtssK (V)
ΔtssK (C)
Δfha
Δfha (V)
Δfha (C)
ΔtssG
ΔtssG (V)
ΔtssG (C)
ΔtssF
ΔtssF (V)
ΔtssF (C)
ΔtssE
ΔtssE (V)
ΔtssE (C)
ΔtssC40
ΔtssC40 (V)
ΔtssC40 (C)
ΔtssC41
ΔtssC41 (V)
ΔtssC41 (C)
ΔtssB
ΔtssB (V)
ΔtssB (C)
ΔtssA
ΔtssA (V)
ΔtssA (C)
ΔclpV
ΔclpV (V)
ΔclpV (C)
Δhcp
Δhcp (V)
Δhcp (C)
Δ46
Δ46 (V)
Δ46 (C)
ΔvgrG-1/-2
ΔvgrG-1/-2 (V)
ΔvgrG-1/-2 (C-2)
ΔvgrG-1/-2 (C-1)
T S
Hcp
ActC
15
35

(B)

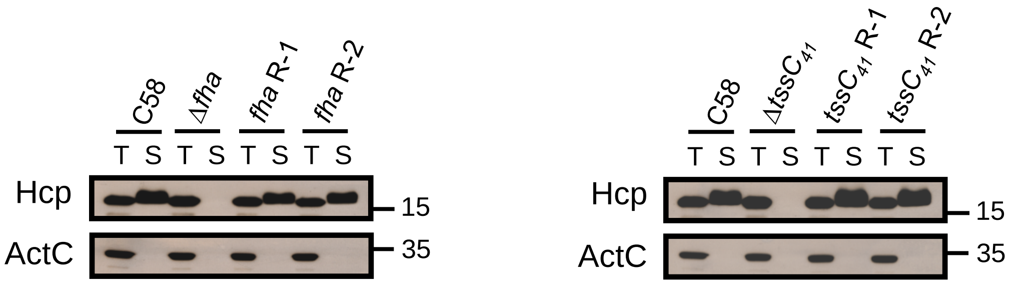
C58
Δfha
fha R-1
fha R-2
ΔtssC41
tssC41 R-1
tssC41 R-2
T S
Hcp
ActC
15
35

Fig. S2

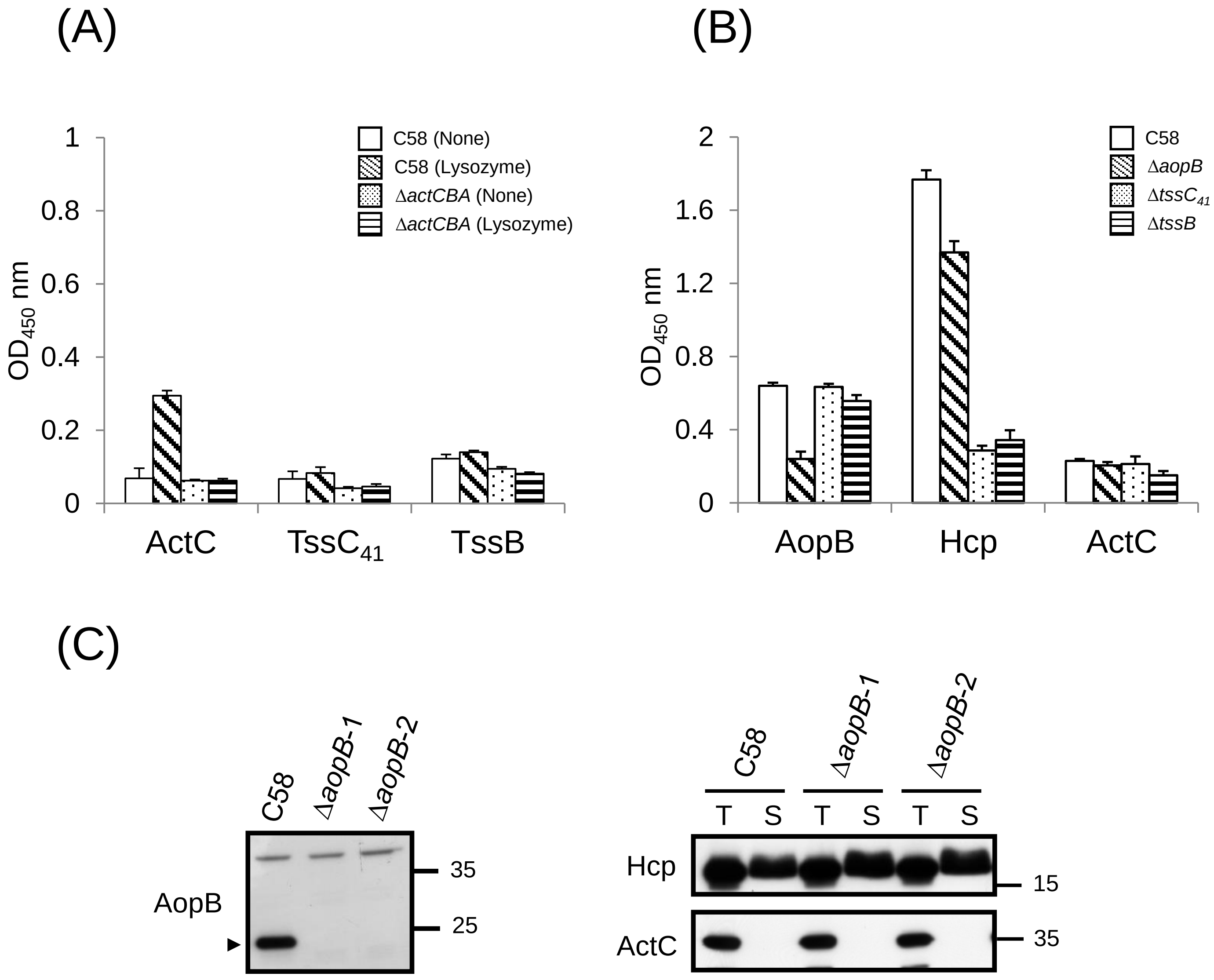
(A)
C58 (None)
C58 (Lysozyme)
ΔactCBA (None)
ΔactCBA (Lysozyme)
OD450 nm
1
0.8
0.6
0.4
0.2
0
ActC
TssC41
TssB
(B)
C58
ΔaopB
ΔtssC41
ΔtssB
OD450 nm
2
1.6
1.2
0.8
0.4
0
AopB
Hcp
ActC
(C)
C58
ΔaopB-1
ΔaopB-2
AopB
35
25
C58
ΔaopB-1
ΔaopB-2
T S T S T S
Hcp
ActC
15
35

Fig. S3

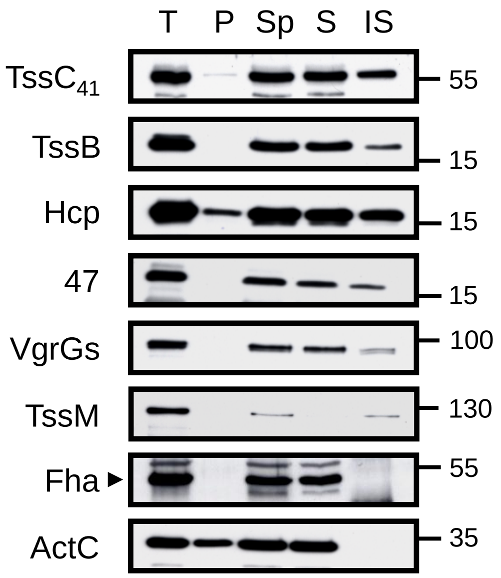
T P Sp S IS
$TssC_{41}$
TssB
Hcp
47
VgrGs
TssM
Fha
ActC
55
15
15
15
100
130
55
35

Fig. S4

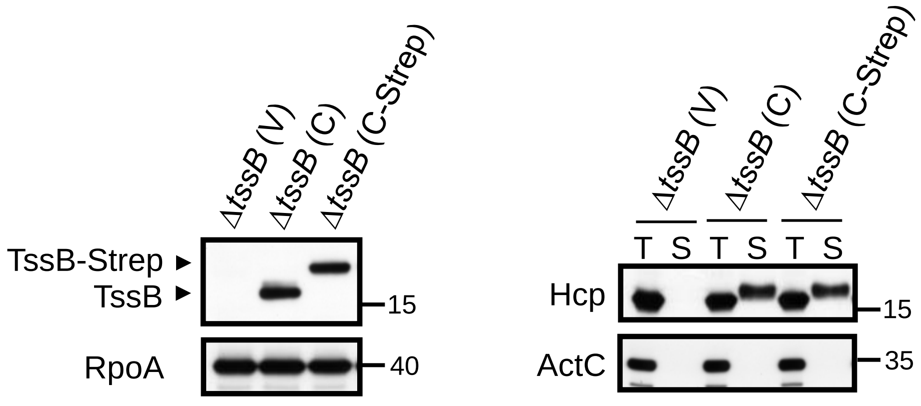

ΔtssB (V)
ΔtssB (C)
ΔtssB (C-Strep)
TssB-Strep
TssB
15
RpoA
40
T
S
Hcp
15
ActC
35

Fig. S5

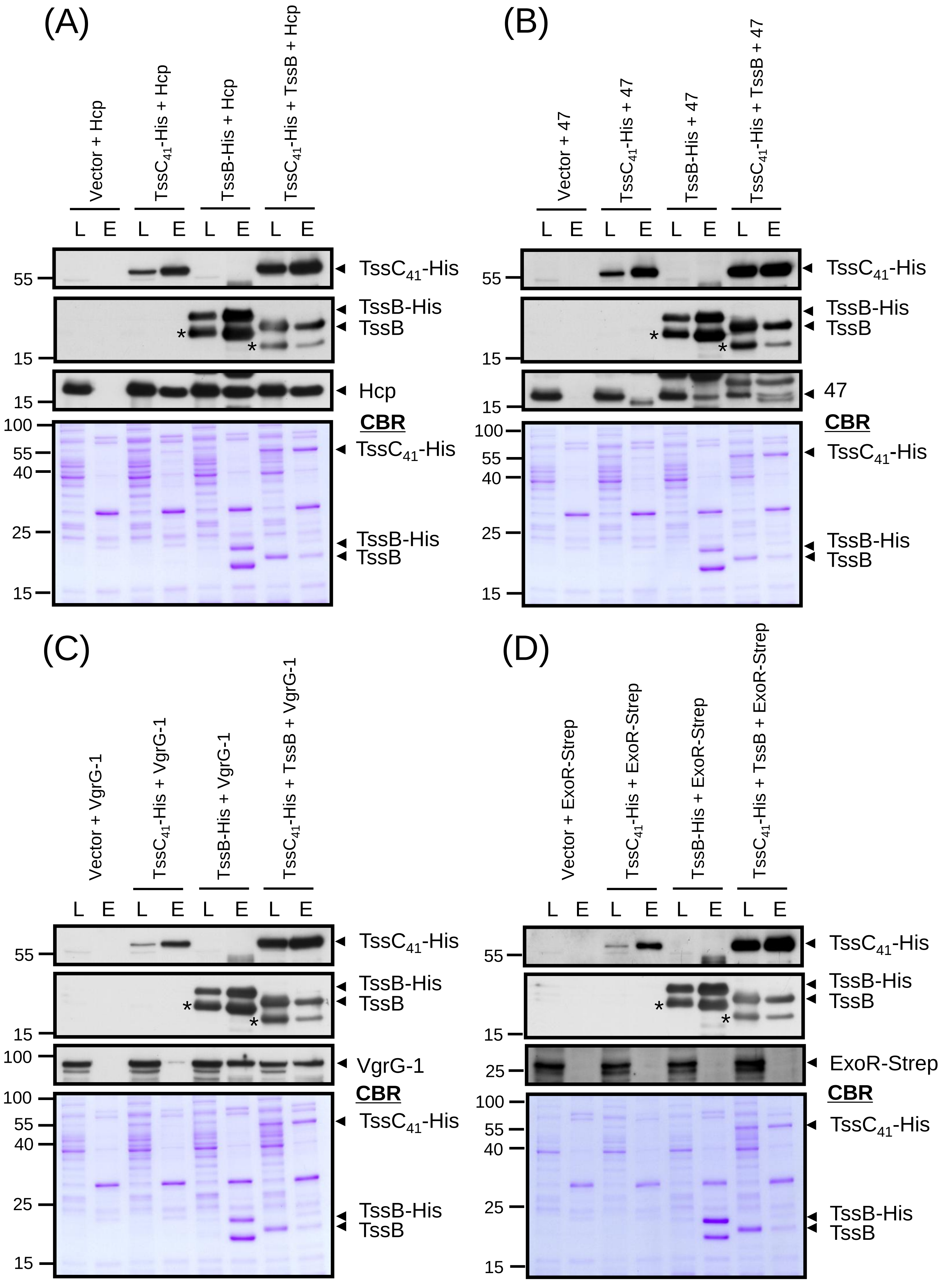
(A)
Vector + Hcp
TssC41-His + Hcp
TssB-His + Hcp
TssC41-His + TssB + Hcp
L E L E L E L E
TssC41-His
TssB-His
TssB
Hcp
CBR
TssC41-His
TssB-His
TssB
100
55
40
25
15
(B)
Vector + 47
TssC41-His + 47
TssB-His + 47
TssC41-His + TssB + 47
47
(C)
Vector + VgrG-1
TssC41-His + VgrG-1
TssB-His + VgrG-1
TssC41-His + TssB + VgrG-1
VgrG-1
(D)
Vector + ExoR-Strep
TssC41-His + ExoR-Strep
TssB-His + ExoR-Strep
TssC41-His + TssB + ExoR-Strep
ExoR-Strep
